# Effectiveness of workplace interventions with digital elements to reduce sedentary behaviours in office employees: a systematic review and meta-analysis

**DOI:** 10.1186/s12966-024-01595-6

**Published:** 2024-04-19

**Authors:** Iris Parés-Salomón, Anna M. Señé-Mir, Fernando Martín-Bozas, Bette Loef, Alan Coffey, Kieran P. Dowd, Guillem Jabardo-Camprubí, Karin I. Proper, Anna Puig-Ribera, Judit Bort-Roig

**Affiliations:** 1 Sports and Physical Activity Research Group, Institute for Research and Innovation in Life and Health Sciences in Central Catalonia (Iris-CC) and University of Vic - Central University of Catalonia (UVic-UCC), Vic, Spain; 2 Sports and Physical Activity Research Group, Sport and Physical Activity Studies Centre, University of Vic - Central University of Catalonia (UVic-UCC), Vic, Spain; 3 Facultad de Educación, Universidad de Zaragoza, Zaragoza, Spain; 4 Center for Nutrition, Prevention and Health Services, National Institute for Public Health and the Environment, Bilthoven, The Netherlands; 5 SHE Research Group, Department of Sport and Health Sciences, Technological University of the Shannon, Athlone, Ireland; 6 Sports and Physical Activity Research Group, Institute for Research and Innovation in Life and Health Sciences in Central Catalonia (Iris-CC) and Faculty of Health Science at Manresa, University of Vic-Central University of Catalonia, Manresa, Spain; 7 Department of Public and Occupational Health, Amsterdam UMC, Vrije Universiteit Amsterdam, Amsterdam Public Health Research Institute, Amsterdam, The Netherlands

**Keywords:** Sedentary behaviour, Workplace, Home-office, Office work, Teleworking, Technology

## Abstract

**Background:**

Digital interventions are potential tools for reducing and limiting occupational sedentary behaviour (SB) in sedentary desk-based jobs. Given the harmful effects of sitting too much and sitting for too long while working, the aim of this systematic review and meta-analysis was to examine the effectiveness of workplace interventions, that incorporated digital elements, to reduce the time spent in SB in office workers.

**Methods:**

Randomised control trials that evaluated the implementation of workplace interventions that incorporated digital elements for breaking and limiting SB among desk-based jobs were identified by literature searches in six electronic databases (PubMed, Web of Science, Scopus, CINAHL, PsycINFO and PEDro) published up to 2023. Studies were included if total and/or occupational SB were assessed. Only studies that reported pre- and postintervention mean differences and standard deviations or standard errors for both intervention arms were used for the meta-analysis. The meta-analysis was conducted using Review Manager 5 (RevMan 5; Cochrane Collaboration, Oxford, UK). Risk of bias was assessed using the Standard Quality Assessment Criteria for Evaluating Primary Research Papers from a Variety of Fields QUALSYST tool.

**Results:**

Nineteen studies were included in the systematic review. The most employed digital elements were information delivery and mediated organisational support and social influences. Multicomponent, information, and counselling interventions measuring total and/or occupational/nonoccupational SB time by self-report or via device-based measures were reported. Multicomponent interventions were the most represented. Eleven studies were included in the meta-analysis, which presented a reduction of 29.9 (95% CI: -45.2, -14.5) min/8 h workday in SB (overall effect: Z = 3.81).

**Conclusions:**

Multicomponent interventions, using a wide range of digital features, have demonstrated effectiveness in reducing time spent in SB at the workplace among desk-based employees. However, due to hybrid work (i.e., work in the office and home) being a customary mode of work for many employees, it is important for future studies to assess the feasibility and effectiveness of these interventions in the evolving work landscape.

**Trial registration:**

The review protocol was registered in the Prospero database (CRD42022377366).

**Supplementary Information:**

The online version contains supplementary material available at 10.1186/s12966-024-01595-6.

## Introduction

Recent advancements in technology have led to a significant increase in sedentary behaviour (SB) in the workplace [1]. Office workers who have a desk-based occupation spend the majority of their daily time (68%) in workplace sitting [2, 3]. High levels of workplace SB has a significant impact on employees’ physical and mental health, along with work-related outcomes, such as work performance and presenteeism [4–7]. Moreover, breaking up prolonged sedentary periods and replacing them with physical activity (PA) of any intensity has been shown to provide health benefits [8–10]. Given that work is the primary domain where SB commonly occurs in office workers, it is crucial to prioritise interventions that target this behaviour to improve desk-based workers’ health, as well as work-related outcomes [8, 11–14].

Several systematic reviews have been conducted in recent years to assess workplace interventions targeting SB [15–17]. These studies, including 34 [15], 26 [16] and 40 [17] studies respectively, have described a wide variety of interventions, including physical changes in the workplace design and environment (e.g., sit-stand desks), policies to change the organisation of work (e.g., breaks to sit less), provision of information and counselling (e.g., distribution of leaflets), and multicomponent interventions [15]. The interventions reviewed, rating the quality of evidence of the most included studies as low or very low [15], fair [16] or non-reported [17], demonstrated a broad range of levels of effectiveness on SB measured by self-reported or via device-measures. However, none of them focused on examining what specific elements of the intervention were most effective. Additionally, many of the interventions required substantial investment (i.e., sit-to-stand desks), while the effectiveness of more cost-efficient and scalable interventional approaches, such as digital interventions [18], were not determined.

Recent evidence has highlighted the potential of technology to enhance behavioural change interventions [19], especially to promote PA and reduce SB [20]. A scoping review classified the digital features that may help to reduce SB among office workers, such as information delivery, digital log, passive data collection, connected device, scheduled prompts, automated tailored feedback, and mediated organisational support and social influences [21]. However, to our knowledge, no previous reviews have analysed the effectiveness of workplace digital interventions to reduce time spent in SB in office workers as the target population.

In this context, it is essential to acknowledge the technological elements that have the potential to facilitate workplace interventions to influence employees’ behaviours. Therefore, the aim of this systematic review and meta-analysis was to examine the effectiveness of workplace interventions that incorporated digital elements to reduce SB in office workers.

## Methods

The current systematic review was performed following the Preferred Reporting Items for Systematic Reviews (PRISMA) guidelines. The review protocol was registered in the Prospero database (CRD42022377366).

### Search strategy

Six electronic databases (PubMed, Web of Science, Scopus, CINAHL, PsycINFO and PEDro) were searched for relevant articles published from 2017 (date of the most recent studies included in the last review on the topic) to October 2023. The reference lists of the included studies were then reviewed. The search included terms related to office work, SB, and digital technology (Table 1).

**Table 1:** Search strategy

	**Subject heading 1:** Office work		**Subject heading 2:** Sedentary Behavior		**Subject heading 3:** Technology
Synonyms and related topics (OR)	“Office work” OR “office employees” OR “computer-based work” OR “screen-based work” OR “seated posture” OR “chair” OR “desk work” OR “white-collar” OR “workplace” OR “worksite” OR “job” OR “occupation” OR “teleworking” OR “telework” OR “remote working” OR “flexible workplaces” OR “home office”	AND	“Sedentary” OR “sedentary behavior” OR “sedentary breaks” OR “sitting” OR “screen time” OR “computer time” OR “sedentary time”	AND	"Digital” OR “internet” OR “web” OR “smartphone” OR “mobile phone” OR “cell phone” OR “mobile application” OR “wearable” OR “technology” OR “software” OR “computer” OR “internet of things” OR “media” OR “email” OR “sensor” OR “activity tracker” OR “eHealth” OR “mHealth” OR “telemedicine”

### Eligibility criteria

Eligible study designs included, randomised controlled trials (RCTs), crossover RCTs, cluster-RCTs, and quasi-RCTs. The Population, Intervention, Comparison, and Outcomes (PICO) characteristics were: office workers (i.e., ≥ 18 years) whose occupations involved spending most of their working time sitting at a desk; a digital element as part of the intervention to reduce SB (i.e., mobile technologies, computers software, messages, wearable devices such as activity trackers for self-monitoring activity patterns, providing feedback or prompts, social media, or websites for improving health, sharing experiences, changing perceptions and cognitions around health, assess and monitoring SB); against a control, comparison and/or other intervention group; and duration of time spent in SB during working hours or on work days measured either by self-report or using device-based measures.

### Study selection

Initially, a single reviewer (FMB) screened titles and abstracts for inclusion. Duplicates were eliminated using reference management software (Zotero, Corporation for Digital Scholarship, George Mason University). Full texts of the remaining articles were independently assessed by two researchers (FMB, IPS), and in case of any disagreements, a discussion with a third reviewer (JBR) took place.

### Data extraction

For selected articles, the following data were extracted: article characteristics (i.e., authors, year, and country), study population (i.e., job type, age, gender, and sample size), study design, intervention characteristics (i.e., type of intervention, general description including the dose and theoretical basis if used, duration and digital features), SB measurement tool (i.e., self-report or device-based measures), primary and secondary outcome measures, and main statistical findings (Table 3). The type of intervention was classified into four categories: physical changes in the workplace design and environment (e.g., height-adjustable desk), policies to change the organisation of work (e.g., active breaks), provision of information and counselling (e.g., educational e-booklet), and multicomponent interventions (i.e., combining at least two of the three above) [15]. Digital elements (i.e., information delivery, digital log, passive data collection, connected device, scheduled prompts, automated tailored feedback, and mediated organisational support and social influences, see Table 2) of the interventions were also documented specifying what digital element of the intervention covers each category [21]. The outcome extracted was time spent in SB at work or in a working day. For missing information, corresponding authors were contacted by email using a template. One reviewer (IPS) extracted the relevant information, and a second reviewer (JBR) checked/confirmed the data.

**Table 2:** Digital elements description

Digital element	Definition
Information delivery	Variation of digital media (text, videos) used to present static information over time (e.g., health facts, motivational messages, tips, suggestions).
Digital log	Users’ entering data through a digital media (e.g., mobile phone diary for self-monitoring, web-based questionnaires)
Passive data collection	Automatic SB or PA records obtained through wearables, smartphones, computer software’s or other technological methods
Connected device	External sensor devices connected to central computing device through wirelessly or with a cable.
Scheduled prompts	Reminders for breaking SB delivered either at fixed intervals or with some schedule adaptive to the real-time users’ status.
Automated tailored feedback	Feedback on individual behaviours and goals or challenges progress, which require data calculations from digital log or passive data collection.
Mediated organisational support and social influences	Messages conveying managers’ approval, users’ communication and/or competition through digital elements, such as online forums for the social influences or organisational support purpose.

### Data analysis

The meta-analysis was conducted using Review Manager 5 (RevMan 5; Cochrane Collaboration, Oxford, UK) and following the general recommendations in the Cochrane handbook for Systematic Reviews of Interventions [22].

The adjusted mean difference (AMD) and standard deviation (SD) of the intervention and control groups were extracted for studies reporting these measures. For studies that reported the AMD and 95% confidence interval (CI) instead of SD, the AMD was extracted, and the standard error (SE) was calculated, which was entered in RevMan 5 to calculate the SD. For studies that did not report the AMD, the unadjusted mean difference (UMD) was calculated from the means at baseline and postintervention in each group. For missing information, authors were contacted via email.

The mean differences were combined using time spent in SB in minutes per eight-hour workday (min/8 h workday) as a standard unit as this was the most prevalent unit presented in the included interventions. Studies which reported min/8 h workday were combined with studies which reported other units, such as hours per week, hours per workday or minutes per day. The latter units were firstly converted to minutes if this was necessary, and then scaled from week to day, and subsequently converted to min/8 h, considering a day as 24 h or a workday as 8 h. One study presented SB in minutes per shift, the shift was assumed as eight hours. Studies with multiple intervention arms were included as two separate studies, while studies with multiple time points, the baseline and “postintervention” measures (collected at the end of the intervention) were included as one study in the meta-analysis, no follow-up measures outside the specified intervention time were used. The sensitivity of the pooled intervention effects was assessed. The overall combined intervention effect was estimated using the random effect model and the inverse variance. Heterogeneity was assessed by I^2^, and significance was set at *p* < 0.05. Inverse variance weighting was used to compensate for heterogeneity of sample sizes between studies. The sensitivity of the pooled intervention effects was assessed after the exclusion of one study through the leave-one-out method.

The meta-analysis was performed for all studies together and for the following subgroups: 1) studies that applied device-based measures for measuring SB, 2) studies that compared a workplace intervention that included digital elements with another workplace intervention that included digital elements, 3) studies that compared a workplace intervention that included digital elements with a usual care group, and 4) studies in which the core elements of the intervention were digital. Additionally, a sub-analysis was conducted comprising of subgroups two and three.

### Risk of bias assessment

Risk of bias was assessed using the Standard Quality Assessment Criteria for Evaluating Primary Research Papers from a Variety of Fields QUALSYST tool. The QUALSYST consists of a 14-item checklist, where every item is scored depending on adherence to the specific criterion (“yes” = 2, “partial” = 1, “no” = 0, and “n/a” = not applicable). Included articles were assessed independently by two reviewers (AC, IPS). Discrepancies were discussed with two additional reviewers (KD, JBR). A summary score was calculated for each paper by summing the total score obtained across relevant items and dividing it by the total possible score.

## Results

### Selected studies

**Fig. 1:**
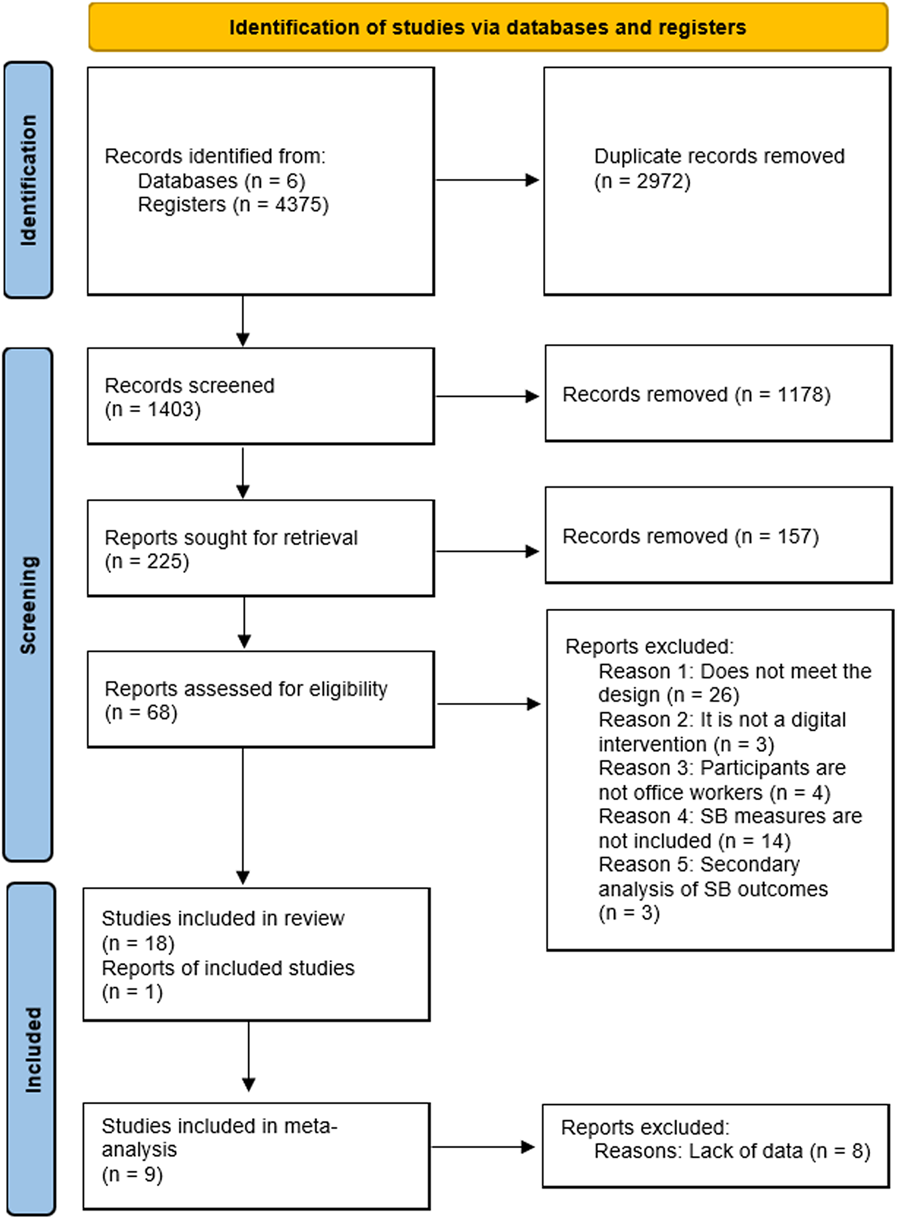
Flow diagram of the study selection process

Figure 1 provides a flow diagram of the article selection criteria for the systematic review. The search in six databases yielded 1403 unique articles. After duplicate review and initial screening of titles and abstracts, 225 full articles were retrieved. A total of 68 full-text articles were critically appraised for eligibility. Fifty articles did not meet the inclusion criteria, and the main reasons were as follows: a) the study design was not a RCT, b) the intervention did not include digital technology features, c) participants were not office workers, and d) the outcome under study did not include SB measures. After reviewing the reference lists of the included studies, one additional article was selected for inclusion in the systematic review [23]. A total of 19 studies were included in the qualitative synthesis.

### Characteristics of the studies

The 19 included studies, which are summarised in Table 3, comprise cluster-RCTs (*n* = 10) [24–33], RCTs (*n* = 5) [23, 34–37], crossover-RCTs (*n* = 2) [38, 39], and quasi-RCTs (*n* = 2) [40, 41]. Studies evaluating workplace interventions that included digital elements to reduce SB ranged from 2 weeks to 12 months in duration. The most common duration of included interventions was between 8 and 13 weeks (*n* = 11) [26, 27, 29, 30, 32–34, 36, 38, 40, 41]. Of the 19 studies, nine included an intervention and a control group (i.e., no intervention elements) [24, 26, 27, 30, 33, 37–39, 41], five included an intervention group and a comparison group (i.e., lighter variant intervention than intervention group) [25, 28, 29, 31, 32], and two included three groups, intervention, comparison and control [25, 31]. Four studies included two intervention groups [23, 34, 35, 40], and one of them also had a third control group [40].

**Table 3:** Characteristics of the studies

Author, Year and Country	Participants and sample size	Study design	Intervention characteristics				Outcomes (measuring tool)	Main findings
			**Type**	**General description**	**Duration**	**Digital features**		
Blake, H., et al. 2019 China [33]	Office workers (52.5% male; nr) *n* = 282	Cluster-RCT	Multicomponent intervention	IG: Website educational materials, instructors’ feedback and videoclips demonstrating Quigong exercises twice per day (10 min). Each video, posted sequentially (i.e., one every two weeks), represented one module, such as neck, shoulder, or arms with three exercises that should be repeated three times. An icon on computer screen was scheduled to pop up twice a day to perform an exercise group led or ignore the prompt and participate individually guided by a video at a time of their preference. The intervention was based on the BCW approach. CG: Control.	12 weeks	SP: Computer screen for popping up. ID: Website educational materials and videos demonstration.	Workday SB (ad hoc survey)	*SB:* It increased in the CG (10.34 ± 1.04 h/week) and in the IG at a lower (5.68 ± 0.82 h/week) from BL to postintervention. The difference in changes (-4.66 h/week) was statistically significant.
Blake, H., et al. 2017 UK [34]	Office workers (86% female; 38.78 ± 10.25 years) *n* = 296	RCT	Information, and counselling	Both groups had access to a website containing educational materials. Four different types of activity-prompting personalized messages of attitudes, subjective norms, perceived behavioral control or intention regarding PA were delivered via email (IG1) or via SMS (IG2) twice a week, at the same time, on the same day, each week. The messages were targeted, tailored and based on the TPB.	12 weeks	SP: Activity-prompting messages. ID: Website educational materials.	Daily SB (GPAQ)	*SB*: Nonsignificant changes for the number of hours per day of SB over time, however, it was lower in both groups at 6 (IG1: 5.47 ± 3.07 and IG2: 5.45 ± 3.83 h/typical day) and 12 weeks (IG1: 5.75 ± 3 and 5.19 ± 3.10 h/typical day) than at BL (IG1: 6.14 ± 3.64 and IG2: 6.23 ± 3.84 h/typical day).
Bort-Roig, J., et al. 2020 Spain [29]	Office workers (82% female; 45 ± 9 years) *n* = 141	Cluster-RCT	Multicomponent intervention	IG: W@W-App included: a) real-time data and self-monitoring of occupational activity; b) visual and auditive prompts with an emoji of a chair that changes according to the time spent in sitting bouts; c) daily summary message by sending data from the app to a web server, which send the message, and weekly motivational messages; d) automated strategies and goals to sit less and move more at work. CG: A partial W@W-App was installed only including the self-monitoring features.	13 weeks	PDC and ID: Mobile phone app for outcome self-monitoring. CD, PDC and SP: Prompts based on a mobile phone app data. ID: Automated strategies and goals. Weekly motivational messages. ATF and PDC: Daily summary message via website.	Total, working, nonworking, workday and weekend SB (activPAL)	*SB:* At post intervention no total SB time changes were found. Differences pre – post intervention during weekday was -0.04 ± 1.62 h/day in the IG and -0.31 ± 1.65 h/day in the CG. Differences between groups were only identified during weekends for SB.
Carter, S.E., et al. 2020 UK [38]	Office workers (57.1% female; 42.5 ± 10 years) *n* = 18	Acceptability and feasibility crossover-RCT	Multicomponent intervention	IG: Educational e-booklet given once and computer-based software to prompt visually every 45 min to interrupt prolonged SB with brief walking activity, when it was activated, it displayed across the whole computer screen and could not be shut down, except during important occasions. The break duration was self-selected, and it was logged automatically in the software after the break. The prompt could be engaged or postponed for 15 min maximum, after that time, the software was automatically activated. Participants received a weekly email with activity feedback and motivation. The intervention was based on the habit formation theory. CG: Control.	8 weeks	PDC, CD and SP: Computer software prompts, automatically activated after 15 min of postpone. ID: Educational e-booklet. Weekly motivational email. ATF and ID: Weekly email with feedback.	Workday, work, and weekend SB (activPAL)	*SB:* Large effects were observed in minutes of workday SB (-38.2 ± 72.9 min/8 h) and the percentage of work SB (-7.7 ± 15.0% work hrs) in favour of IG. Pre – post intervention within-group differences were 26.3 ± 43.7 min/8 h workday in the CG and -11.9 ± 43.2 min/8 h workday in the IG.
Danquah, I. H., et al. 2017 Denmark and Greenland [30]	Office workers (66% female; 46 ± 10 years) *n* = 317	Cluster-RCT	Multicomponent intervention	IG: Social support (from ambassadors and management through appointments); environmental changes (sit-stand desk, hight meeting tables, and routes for walking meetings); informative session and materials (a 15-min lecture to increase knowledge of SB and health and communicated in a leaflet and via website); a workshop guided through four strategies (using sit-stand desk, breaking prolonged SB, having standing and walking meetings, and setting office-level common goals); and volunteer weekly emails and/or twice weekly text messages with regular updates and tips. The intervention was developed using social cognitive theory, Rogers’ diffusion of innovations theory and goal-setting theory. CG: Control.	3 months	ID: Informative materials in a website. Volunteer weekly emails and weekly text messages.	Working, nonworking, and total SB (ActiGraph)	*SB:* Participants at the IG showed 71 min (-85 to -57) less SB per 8 h workday than CG after one month, at 3 months the difference was smaller. No differences between IG and CG were shown in nonwork SB. BL SB in the CG were 334 ± 5.1 min/8 h workday and in the IG, it was 345 ± 4.4 min/8 h workday. Postintervention SB in the CG were 352 ± 5.6 min/8 h workday and 292 ± 5.8 min/8 h workday in the IG.
De Cocker, K., et al. 2017 Belgium [31]	Office workers (68.5% female; 40.3 ± 9.1 years) *n* = 213	Cluster-RCT	Information, and counselling	IG: Web-based computer-tailored advice including tips on how to reduce and/or interrupt workplace sitting and personalized feedback messages on the frequency of interruptions, PA levels, SB were given after completing a questionnaire. Predefined decision rules were used to give tailored feedback messages. An action plan with personalized goals was developed for motivated users. The intervention includes constructs based on the TPB and Self-Regulation Theory. CGs: Generic condition (i.e., nontailored advice on the importance of reducing and interrupting workplace SB), and control condition (i.e., wait-list control).	1 month	ID, DL and ATF: Web-based computed-tailored advice including tips and outcomes feedback based on questionnaire answers. Action plan to motivate participants.	Work SB (WSQ)	*SB*: Completing an action plan was successful in reducing self-reported workplace sitting from 332.8 ± 124.6 min/day to 282.5 ± 105.3 min/day, while this results there were not show in the CGs (353.3 ± 128.1 min/day in the generic condition and 284.6 ± 61.3 min/day in the control condition at preintervention and 351.8 ± 128.4 and 283.5 ± 60.4 min/day, respectively at postintervention).
Edwardson, C.L., et al. 2018 UK [24]	Office workers (79% female; 41.2 ± 11.1 years) *n* = 146	Cluster-RCT	Multicomponent intervention	IG: Organisational support meeting with the chief executive, who send a regular e-newsletter and allowing time for intervention activities and encouraging involvement. Electric adjustable height desk or desk platform. 30-min education seminar about health consequences of SB and benefits of breaking it, leaflet containing the same information, action plan and goal setting booklet. Posters with educational or motivational messages every few months. Participants received outcomes feedback after each follow-up data collection and were encouraged to set a goal and to create an action plan. DARMA cushion, which sync data with a mobile phone app prompted by vibration every user defined time (e.g., every 30 or 45 min) and provided real-time feedback. 15-min face-to-face coaching sessions at month 1 and every 3 months to review action plans and give support. The intervention was grounded in several behaviour change theories: social cognitive theory, organisational development theory, habit theory, self-regulation theory, and relapse prevention theory. Additionally, it was implemented through the BCW and the associated COM-B approach. CG: Health measures results were given.	12 months	PDC, CD and SP: DARMA cushion sync with an app to prompt. ID and MOSSI: Regular e-newsletter from chief executive. ATF: Real-time feedback.	Work and daily SB (activPAL)	*SB:* After 12 months work SB showed differences between groups -83.28 (-116.57 to -49.98) min/workday in favour of the IG. In addition, after 12 months significant differences between groups were found for 8-h workday: -41.29 (-59.88 to -22.69), mean change from baseline in the CG was 3.78 (-11.23 to 18.78) and -35.21 (-49.12 to -21.31) min/8 h workday in the IG.
Edwardson, C.L., et al. 2022 UK [25]	Office workers (72.4% female; 44.7 ± 10.5 years) *n* = 756	Cluster-RCT	Multicomponent intervention	IG1: SWAL intervention: organisational strategies (i.e., briefing events about the importance of reducing and breaking up SB and its benefits, online/video brief awareness session about benefits of reducing SB, reviewing policies, and encouraging managers to review the layout of office space, and by managers modelling); environmental strategies (i.e., small-scale environmental restructuring, motivational and reminder signs around the office); Individual and group strategies (i.e., an online education session and set an individual goal and an active plan, SB self-monitoring through optional computer prompts, timers and mobile phone apps, encouragement from workplace champions through coaching, competitions and monthly motivational/educational emails). IG 2: SWAL intervention and height adjustable desk, additionally information booklet, guidance, and recommendations. The SWAL intervention was grounded in social cognitive theory, organisational development theory, habit theory, self-regulation theory and relapse prevention theory. Additionally, it includes a ranged of strategies which draw upon the principles of the BCW and the associated COM-B approach. CG: Control.	12 months	ID: Online/video session. Online education session. PDC and SP: Outcome self-monitoring through computer prompts, timer, or app.	Daily, workday, working, and nonworking SB (activPAL and OSPAQ adapted version)	*SB:* IG1 and IG2 sat for 22.2 (-38.8 to -5.7) min/day and 63.7 (-80.1 to -47.4) min/day less than the CG at 12 months. Changes in IG1 and IG2 compared to CG were favourable in SB during work hours at 12 months -13.4 (-29.0 to 2.2) min/work hours, and -57.9 (-73.3 to -42.5) min/work hours, respectively. Within-group differences from BL to follow-up were 2.2 ± 61.1 min/work hours in the CG, -12.8 ± 71 min/work hours in the IG1 and -56.4 ± 85.5 min/work hours in the IG2.
Engelen, L., et al. 2019 Australia [41]	Office workers (54.3% male; 9.5% 26–35 years) *n* = 46	Pilot quasi-RCT	Multicomponent intervention	IG: Welcome email with tips and guidelines to increase standing at work and short informative video. One-hour workshop in the first week about the risks of prolonged SB and benefits of breaking it, focus groups and brainstorming on how to sit less and move more and three site visits. Sit-stand desks with computer visual and audible prompts to change desk position, increasing the standing time every session. Feedback on the standing and sitting time were provided at the end of the day allowing self-monitor. Weekly emails with strategies and videos. CG: control.	13 weeks	PDC, ATF and SP: Computer prompts and feedback on standing and sitting time. ID: Email with tips and guidelines. Weekly emails with strategies and videos.	Workday, and work SB (ActivGraph)	*SB:* After 6 weeks SB during the workday decreased to 63% in the IG, and after 13 weeks this decrease was sustained, 80%. This equates to approximately 80 min less sitting over an 8-h workday. While in the CG the decrease of workday SB was 79% at 6 weeks and 80% at 13 weeks.
Invernizzi, P. L., et al. 2022 Italy [36]	Office workers (60% female; IG: 31.7 ± 8.2 years, CG: 32.0 ± 4.4 years) *n* = 45	RCT	Multicomponent intervention	IG: Environmental changes including PA workstations (e.g., meeting rooms’ bike), movement during active pauses or during workflow were used three times a week, while twice a week participants worked in smart working. A mobile phone application connected to the workstations using QR codes permitting access to the description of each exercise, the suggested time and to the timer attached to the selected activity. After stop the activity the PA feedback was automatically shown. The app assigned an individual PA goal to be reached during the week. Benefits of PA and healthy lifestyles were explained. Twice a week the correct execution of the exercises (online and in person) was demonstrated. The intervention followed the self-determination theory. CG: Three times a week worked in the usual office and twice in the smart working, as usual.	8 weeks	PDC, CD and ID: Mobile phone app connected to workstation, which provide exercise description. ATF: Mobile phone app feedback. ID: Exercise correct execution explanation.	Workday and weekend SB (Axivity Ax3 and IPAQ)	*SB*: No differences were found in both groups for self-reported and device-based SB measures. IPAQ measures showed working day SB at BL of 506.8 ± 166.1 Met in the IG and 488.1 ± 150.3 Met in the CG, and after intervention workday SB was 432.5 ± 188.0 Met in the IG and 450 ± 137.4 Met in the CG. Accelerometer data at BL were 2334.5 ± 321.4 in the CG and 2442.2 ± 252.6 in the IG, at 8 weeks data change to 2317.3 ± 294.7 in the CG and to 2343.9 ± 262.4 in the IG.
Mamede, A., et al. 2021 Netherlands [32]	Office workers (62.4% female; 46.25 ± 9.8 years) *n* = 256	Cluster-RCT	Multicomponent intervention	Both groups received Fitbit accelerometers linked to an app. Default daily step count goal, and weekly personalized feedback via email on steps number. IG: Virtual walking tour challenges during first 5 weeks (2 challenges lasting 2 weeks with 1 week in between them), including gamification, social support, and comparison features (i.e., team graded challenges with leader board, each one represented a charity, points and regards could win), biweekly newsletter via email. Physical nudges for the last 5 weeks. (i.e., table signs to motivate and remind participants to engage in PA and reduce SB) CG: Basic version of the app. 10000 steps were the daily step goal during the 10 weeks.	10 weeks	PDC and CD: Fitbit accelerometer linked to an app. ID, ATF and PDC: Weekly outcome feedback via email and gamification. PDC: Virtual walking tour challenges. MOSSI: Digital app challenges incorporating social support and social comparison features.	Work SB (2-item self-report measures)	*SB:* No association was found between the intervention condition or study phase and the time spent in SB. However, IG showed SB reductions during work time from BL to follow-up, 30.9 ± 10.7 and 30.0 ± 6.8 h/week, respectively, while CG increase SB from 29.1 ± 10.2 to 29.9 ± 10.7 h/week.
Martin, A., et al. 2017 UK [35]	Sedentary workers (100% male; 65% 30–49 years) *n* = 40	Feasibility and acceptability RCT	Multicomponent intervention	Both groups received SitFIT device, with real-time self-monitoring of daily step accumulation and SB. A colour-coded bar represented the percentage of daily time spent in SB (yellow) and upright time (green). Tactile feedback and vibration prompts after 15, 30 and 45 min of SB, with one, two or three vibrations, respectively. Feedback on periods of sitting uninterrupted. Informative booklet. Three incremental behavioural goals (i.e., increasing daily steps by 1500, reducing SB/increase upright time by 30 min, reducing percentage daily SB by 5%). IG1: Feedback on step count, sedentary time, and percentage sedentary time. IG2: Feedback on step count, upright time, and percentage sedentary time.	4 weeks	PDC, SP and ID: Real-time self-monitoring of outcomes and prompts. ATF: Feedback.	Daily SB (activPAL)	*SB:* At 4 weeks, between-group difference from BL to 4 weeks was 15.2 (-81.6 to 112.0) min/day of SB, while at 12 weeks this difference was -64 (-160.7 to 32.8) min/day of SB. From BL to 4 weeks within-group differences in IG2 were -4.6 (-75.4 to 66.3) and in IG1 the difference was -11.1 (-81.3 to 59.1) min/day of SB.
Maylor, B. D., et al. 2018 UK [26]	Office workers (57% female; 43.47 ± 12.47 years) *n* = 90	Cluster-RCT	Multicomponent intervention	IG: Educational and brainstorming sessions to identify strategies and about on the dangers of prolonged SB, which were then emailed. Participants were provided with a pedometer. Step challenges during the entire intervention period. Daily steps were entered onto a virtual leader board and spot prizes were provided. 20-min health check with the report and informative material. Prompts from computer software and/or phone app and visual support (i.e., poster prompts). Weekly telephone support (from week 2 to 8). Work environmental changes (e.g., relocation of printers). CG: control.	8 weeks	DL: To enter outcome onto a virtual leader board. CD: Pedometer supplemented with computer software prompts. SP: Computer and/or phone app prompts. MOSSI: Weekly telephone support and step challenges.	Daily and work SB (activPAL)	*SB:* There was no significant difference between IG and CG in SB change at work -15.7 (-38.0 to 6.5) min/shift. At 8-week IG reduced SB by 15.7 (-35.7 to 4.3) min/shift, while CG increase this time (0.9 (-20.6 to 22.5) min/shift). No significant differences were found in daily sitting time.
Morris, A. S., et al. 2020 UK [40]	Office workers (64% female; 39.8 ± 11.4 years) *n* = 56	Feasibility quasi-RCT	Multicomponent intervention	IG: Smartphone application for iOS devices. Two break frequencies, 30 min (IG1) or 60 min (IG2), the duration of break was not prescribed. The app notified the breakthrough sound and/or vibration and pop-up notification (“time to stand up”), the breaks were manually entered. The app allowed participants to self-monitor. The intervention was aligned with the intrapersonal level within the socioecological model. CG: Smartphone application for android devices, to self-monitor the SB breaks. The application did not provide prompts, and the breaks were manually entered.	12 weeks	DL: Breaks manually entered. SP and ID: Pop-up notifications and self-monitoring through an app.	Work SB (activPAL)	*SB:* Significant difference between groups, in favour of IG2 was observed for total worktime SB at 12 weeks, relative to CG -69.8 (-111.0 to -28.2) min/8 h workday. Relative to the CG, there were changes in worktime SB in IG1 at 12 weeks -37.0 (-78.0 to 4.2) min/8 h workday, although these were not statistically significant. At BL worktime SB was 329.4 ± 83.1 in the CG, 320.7 ± 110.5 in the IG1 and 345.2 ± 67.8 min/8 h workday in IG2, at 12 weeks this time changed to 369.5 ± 86 in the CG, to 326 ± 119.3 in the IG1 and to 302.4 ± 117.4 min/8 h workday in the IG2.
Nicolson, G. H., et al. 2021 Ireland [39]	Office workers (100% male; 42.9 ± 11 years) *n* = 21	Pilot feasibility crossover-RCT	Multicomponent intervention	IG: Education session (i.e., the dangers of SB and benefits of its reduction). Under desk pedal and Garmin tracker watch. Cycling time goals of 30–40 min per workday. Manual measurement of pedalling times using the Garmin watch facilitated self-monitoring, and immediate feedback on pedalling time on the Connect platform allowed social comparison, and friendly competition. Weekly email feedback. Visual alert (i.e., move bar) on the Garmin watch every 15 min of SB, which accumulated to provide sound and vibration after one hour of SB through, some PA were required to reset it. The intervention was developed applying the socio-ecological model. CG: Control.	2 weeks	DL: Manual measurement of pedalling time. PDC and SP: Garmin watch prompts every 15 min of SB. CD: Garmin watch connected to a Connect platform. ATF: Feedback on pedalling time. ID, PDC, and MOSSI: Social comparison and friendly competition through a Connect platform.	Daily and work SB (activPAL)	*SB:* It was shown a decrease in workday SB from 379.3 ± 79.0 to 358.9 ± 96.9 min/workday in the IG compared to CG, thus, an indicative reduction of workplace SB of 20.4 min/workday. Total weekday SB was reduced by 45.7 min/day in the IG compared to the CG.
O'Dlan, C., et al. 2018 UK	Office workers (76% female; 39 ± 8.5 years) *n* = 19	Feasibility cluster-RCT	Information, and counselling	Both groups received an educational session (health risks associated with SB, potential benefits, and tips to break SB). IG: Seventy brief, positively and with the organisation name framed messages were prompted on the screen, one of which was sent a half period in the middle of each hour through Microsoft Outlook. CG: Control.	10 weeks	SP, ID, and MOSSI: Organisation signed prompt messages.	Daily and work SB (activPAL)	*SB:* Between group differences showed a lower proportion of time spent in SB during working hours in all the measures in favour of the IG. At BL total sitting work hours in the IG was 71.8 ± 22% and in the CG this time was 78.7 ± 11.8%, at 8 weeks this time changed to 69.4 ± 17.2% and 72.2 ± 15.0%, respectively.
Pereira, M., et al. 2020 USA [23]	Office workers (74.4% female; 44.6 ± 11.2 years) *n* = 630	RCT	Multicomponent intervention	Both groups received strategies based on the socio-ecological framework, including policy-level components (i.e., a leader and advocate, 5-min break every hour, and four quarterly support emails sent by the leader of employees,), environmental changes (i.e., walking routes, footrests, and other changes implemented by leader and advocate, such as challenges), individual and social components (i.e., weekly e-newsletter (*n* = 26) for 1st month and biweekly from 2nd month, coaching session to identify goals and strategies). All the intervention materials were manualized into Toolkit. IG1: The target was to increase LPA time, replacing 30 min of sit time with movement. IG2: Sit-stand workstation. The target was increasing standing and LPA time, achieving a 30:30-min sit to stand ratio.	12 months	ID: a total of 26 e-newsletters ID and MOSSI: Support emails sent by the leader of employees.	Daily and work SB (activPAL)	*SB:* At 12 months between-group difference was -59.2 (74.6 to -42.8) min/8 h workday. From BL to 12 months, sitting at work was significantly decreased in IG2 compared to IG1: -52.5 (-62.9 to -42.9) min/8 h workday and 6.8 (-4.3 to 17.8) min/8 h workday, respectively.
Rollo, S., et al. 2020 Canada [37]	Office workers (91.7% female; nr) *n* = 60	RCT	Multicomponent intervention	IG: Phone counselling session (coping strategies identification focused on increasing break frequency to every 30–45 min, achieving a break duration of 2–4 min and creating action plans), information booklet and planning sheet. Text messages reminded to review and change action plans at the beginning of week 3 and 5 (3–4 action plans). Daily SB-related text messages, accompanied by tips, challenges, and reminders to reduce SB, intending as mini-booster interventions. Two difficulty gradual challenges each week to break up and reducing SB trying to reduce total SB at work by 2 h or greater. The intervention used the health action process approach. CG: control.	6 weeks	ID and ATF: Text messages reminders and daily text messages about SB. ID and MOSSI: Text messages including challenges.	Work SB (OSPAQ)	*SB:* Relative to BL SB 353.6 ± 80.7 min/workday decreases to 269.4 ± 115.8 min/workday at 6-week in the IG, which were significantly greater at all time points for the IG compared to those in the CG (BL: 358.8 ± 78.3 min/workday, 6-week: 355.8 ± 74.2 min/workday).
Thogersen-Ntoumani, C., et al. 2020 Australia [28]	Office workers (82.50% female; 21–66 ± 10.29 years) *n* = 97	Pilot feasibility cluster-RCT	Multicomponent intervention	IG: 1-h face-to-face workshop, motivational and educational training, and manual (i.e., strategies and behavioural change techniques). iOS app with motivation-supportive communication (i.e., feedback and weekly goal progress, reminders messages) and behavioural change techniques. Fitbit Zip to self-monitoring entering daily step count and recording walks on the app, and a step count goal were advised. Peer leader teams to join lunchtime walks, with a gradually progress to self-organize walks without peer-led team (i.e., 30-min walks twice per week and reduced the peer-led walks to once per week from weeks 7–10; walkers were encouraged to self-organize their own walks 3 times per week, for the last 6 weeks there were no peer-led group walks, walkers were encouraged to engage five self-organized walks per week). CG: Fitbit Zip. To accumulate 7500 steps per day, brief talk and a leaflet.	16 weeks	CD: Fitbit Zip recording data outcomes on the app. PDC, ID, ATF: Motivation-supportive communication through the app (i.e., feedback, self-monitoring, goal progress, and reminder messages).	Daily SB (activPAL)	*SB:* Daily SB decreased in the IG from 9.79 ± 1.18 to 9.43 ± 1.99 h/day at postintervention, while it increased slightly in the CG from 9.84 ± 1.47 at BL to 9.92 ± 1.41 h/day at postintervention.

*RCT* randomised control trial, *IG* intervention group, *BCW* Behaviour Change Wheel, *CG* control group, *PDC* passive data collection, *ID* information delivery, *ATF* automated tailored feedback, *MOSSI* meditated organisational support and social influences, *SP* scheduled prompts, *SB* sedentary behaviour, *UK* United Kingdom, *TPB* Theory Planned Behaviour, *GPAQ* Global Physical Activity Questionnaire, *CD* connected device, *WSQ* Workforce Sitting Questionnaire, *BL* baseline, *DL* digital log, *COM-B* Capability, Opportunity; Motivation and Behaviour

Studies have been undertaken in a wide range of countries. The most represented countries were the United Kingdom (*n* = 8) [24–27, 34, 35, 38, 40] and Australia (*n* = 2) [28, 41]. European countries such as Spain [29], Denmark [30], Belgium [31], Italy [36], the Netherlands [32], and Ireland [39] were also represented.

A total of 3529 participants were included in the 19 studies, with samples sizes ranging from 18 to 756. All the participants were adult office workers, and most of them were women who represented a mean of 61.7% in the included studies. Two studies solely focused on men [35, 39].

### Measurement methods

Occupational and nonoccupational SB outcomes were measured by self-report questionnaires (*n* = 7) [25, 31–34, 36, 37] or via device-based measures (*n* = 14) [23–30, 35, 36, 38–41], with two studies utilising both approaches [25, 36]. Self-reported tools included were the Global Physical Activity Questionnaire (GPAQ) [34], Workforce Sitting Questionnaire (WSQ) [31], International Physical Activity Questionnaire (IPAQ) [36], Occupational Sitting and Physical Activity Questionnaire (OSPAQ) [25, 37], and unvalidated or adapted questions [32, 33]. Fourteen studies employed thigh-based accelerometers, with the activPAL (PAL Technologies, Glasgow, UK) being the most employed (*n* = 10) [23–29, 35, 38–40], and three studies used ActiGraph GT3X + (ActiGraph, Shalimar, FL, USA) [30, 41]; only one study applied a wrist-based accelerometer, the Axivity AX3 (Axivity, Newcastle upon Tyne, UK, 2013) [36].

Table 4 shows the measurement unit of SB, presented in a wide range of ways, such as in hours, minutes, or proportions of time spent in SB during the day, workday, or work hours. Some of the studies reported the data in more than one measurement unit [24, 25, 27, 29, 36, 38–40].

**Table 4:** Measurement units according to the different studies

Measurement unit	Number papers (reference)
**Hours**	
Hours/week	3 [29, 32, 33]
Hours/typical day	1 [34]
Hours/day	2 [28, 29]
Hours/workday	1 [29]
Hours/working time	1 [29]
Hours/nonworking time	1 [29]
Hours/weekend	1 [29]
**Minutes**	
Minutes/8 h workday	5 [23, 24, 30, 38, 40]
Minutes/16 h workday	1 [40]
Minutes/shift	1 [26]
Minutes/day	3 [24, 25, 31, 35]
Minutes/weekday	1 [39]
Minutes/workday	3 [24, 37, 39]
Minutes/work hours	1 [25]
Minutes/day on nonworkdays	1 [25]
Minutes/weekend	1 [39]
**METs**	
Met/working day	1 [36]
Met/weekend	1 [36]
**Proportion (%)**	
Proportion of time spent sitting during work	1 [41]
Proportion of workday sitting	1 [25]
Proportion of total sitting work hours	2 [27, 38]
Proportion total sitting all days	1 [27]

*METs* metabolic equivalents of task

### Digital element of the intervention characteristics

Multicomponent interventions were used in 16 studies [23–26, 28–30, 32, 33, 35–41], seven of which included elements from design and environmental changes (e.g., sit-stand desks) [23–25, 30, 36, 39, 41], and 15 of which comprised policies to change the organisation (e.g., SB breaks) [23–26, 28–30, 32, 33, 35, 37–41]. All of them included information and counselling interventions (e.g., prompts, distribution of leaflets or counselling). Three studies only included information and counselling interventions [27, 31, 34].

All studies combined different digital features. Information delivery was included in 18 of the studies [23–25, 27–41]. Digital media forms of information delivery cover educational and informational materials to increase knowledge and awareness in a range of ways, such as e-booklets [38], e-newsletters [23, 24], website [30, 31, 33, 34], online sessions [25], videos [33, 41], Toolkit [23], Garmin watch [35], and gamification tools [32]. Two of the studies did not specify what type of channel was used to distribute text messages [30, 37]. Text messages sent through emails [23, 30, 32, 38, 41], mobile phone application [28, 29], or computer software [27] were also covered by the information delivery digital element.

Automated tailored feedback (*n* = 11) comprised periodical feedback of the individual or team behaviours and progress, as well as goal accomplishment, sent in a variety of ways (i.e., emails [32, 38, 41], uploaded in a mobile phone application [24, 28, 36], website [29, 31, 39], and visually via the wearable device [35]). One study did not specify what channel was used to send text messages [37].

Scheduled prompts, such as reminders to break SB [24–27, 29, 35, 38–40] and/or to participate in PA [26, 33, 34, 39], as well as to use environmental strategies (i.e., change sit-stand desk position) [41] were implemented in 12 studies [24–27, 29, 33–35, 38–41]. Prompts were delivered visually [29, 33, 34, 38, 39], audibly [29, 39–41] and/or by vibration [24, 35, 39, 40] through computer screens [25–27, 33, 38, 41], emails [34], mobile phone applications [25, 26, 29, 40], SMS [34], wearables (i.e., smartwatches [39], or bracelets [35]) and/or seat cushions [24]. Three studies did not report the delivery method of the reminder [25–27]. The frequency and duration of the prompts and breaks differed in each intervention, being either selected by the intervention administrator or self-selected by the workers themselves.

Ten studies reported passive data collection of time spent in SB through applications [24, 25, 28, 32, 36], websites [29, 39], wearables [35], and software [25, 38, 41]. Seven of them used an external connected device, such as mobile phones [29, 36], computers [38], wearables [28, 32, 39], or cushions [24]. One study combined an external device (i.e., Garmin watch) and a digital log to self-report manual pedalling time [39]. Three studies used a digital self-monitoring log of the behaviour through a mobile phone diary [40], a virtual board [26], and a questionnaire [26, 31, 40].

The mediated organisational support and social influences were represented in seven studies covering e-newsletter from the managers’ [24], support emails from employees’ leader [23], organisation signed prompt messages [27], telephone support [23], and challenges [26, 32, 37] allowing (or not) social comparison [32].

### Effectiveness of the intervention with digital elements in reducing SB

Six out of 16 multicomponent interventions [23, 24, 30, 33, 37, 40], including information delivered through e-newsletters [23, 24], website [30, 33], video demonstrations [33], or text messages [37, 40]; non-digital physical changes (i.e., height adjustable desks [23, 24, 30]); prompts to break SB or participate in PA delivered through a cushion [24], computer screen notifications [33] or mobile phone application notifications [40]; support from the organisation and social influences demonstrated through emails [23], e-newsletters [24], or challenges [37]; feedback on the behaviour [24, 37]; and/or behaviour data collected through a device or manually entered [24, 40], reported significant changes in time spent in SB at work. Ten multicomponent interventions reported reductions, although they were not statistically significant on daily, workday, or working SB [25, 26, 28, 29, 32, 35, 36, 38, 39, 41]. These interventions comprised informational and educational material delivered through online sessions [25], emails or app messages [29, 38, 41], e-booklets [38]; feedback on behaviour [28, 32, 35, 39, 41] collected passively [25, 28, 29, 32, 35, 38, 39, 41] (i.e., through a wearable [28, 32, 39], mobile phone app [29] or computer [38] connected to an application [28, 32], website [29], computer software [38] or platform [39]) or manually [26, 39] (i.e., entering data onto a virtual board [26] or onto a platform [39]); organisational support and social influences illustrated through challenges [26, 32], or social competition [39], prompts delivered through the computer [25, 26, 38, 41], mobile phone app [25, 29] and/or wearables [35, 39]. Only one of them, characterized by a mobile phone application including real-time data and self-monitoring, prompts, daily summary messages and weekly motivational messages, automated strategies and goals, showed higher reductions, but not statistically significant, in the comparison group, which used a partial application including self-monitoring features, compared to the intervention group [29].

Ten of the 16 multicomponent interventions were developed based on theories of behaviour change [23–25, 30, 33, 36–40]. Three of these interventions were grounded in multiple theories [24, 25, 30], while the other studies were based only on one theory. The Behaviour Change Wheel (BCW) [24, 25, 33] and socio-ecological model [23, 39, 40], as well as the habit formation model/theory [24, 25, 38] and social cognitive theory [24, 25, 30] were the most commonly employed theories. Other theories employed for the development of interventions were organisation development theory [24, 25], self-regulation theory [24, 25], relapse prevention theory [24, 25], Roger’s diffusion of innovations theory [30], goal-setting theory [30], self-determination theory [36], and the health action process approach [37]. The six multicomponent interventions, which demonstrated significant changes, used theories to develop their interventions [23, 24, 30, 33, 37, 40].

Two of the three studies that comprised information and counselling interventions, including prompting positive messages on the computer screen [27] and web-based computer-tailored advice, feedback messages and action planning [31], showed higher reductions, although not statistically significant, for intervention groups compared to control groups in time spent in SB during work [27, 31]. One information and counselling intervention, which had two intervention groups and no control group and implemented website educational materials and messages via SMS or email. The two groups showed reductions that were not statistically significant in daily SB at 12 weeks [34]. Two of the three information and counselling interventions were based on theories, such as the theory of planned behaviour [31, 34] and self-regulation theory [31], to develop their interventions. One of the two studies followed two theories to develop the intervention and showed reductions in SB time, but these changes were not statistically significant [31]. One study did not use a theory to develop the intervention, and showed non statistically significant reductions in SB [27].

Four studies had an intervention and treatment active-comparison group, including prompts and feedback on SB time vs no prompts and no feedback [29]; action plan vs no action plan [31]; and different goals vs the same goal across the intervention [28, 32]. One of these studies had three groups: intervention, comparison and control [31]. All the studies showed reductions in SB, but none of them were statistically significant. Additionally, the study with three intervention arms showed higher reductions between the intervention and comparison groups than studies that included two intervention arms. Five studies had two intervention groups, including height-adjustable desk vs no desk [25]; prompts every 30 min vs 60 min [40]; feedback on SB time vs feedback on upright time [35]; increased standing (with height-adjustable desk) vs increased moving time (no desk) [23]; and messages via SMS vs via email [34]. Two of them included three groups, two intervention and one control group [25, 40]. The five studies showed reductions in SB; three of them showed favourable differences between groups in favour of the height-adjustable desk and prompts every 60 min [23, 25, 40], while two others reported higher reductions in intervention groups that included messages via SMS and feedback on SB time [34, 35]. However, in only two of the five studies were these changes statistically significant [23, 40].

Of eleven studies using activPAL as the measurement tool [23–29, 35, 38–40], ten revealed reductions in SB time in intervention groups compared to control groups [23–28, 35, 38–40]. Three of these were statistically significant [23, 24, 40]. Only one study showed higher reductions in the comparison group than in the intervention group [29]. Two studies used the ActiGraph accelerometer as a device-based measure, and both reported higher reductions in SB during work in favour of the intervention groups [30, 41]. One study using Axivitiy as a device-based measure and the IPAQ as a self-reported measure did not find significant differences in either measurement method [36]. Those studies employing the WFQ and OSPAQ showed reductions in SB time in the two groups, with higher reductions in the intervention group [25, 31, 37]. Measuring SB with GPAQ also showed reductions in SB time from baseline to postintervention, although these findings were not statistically significant [34]. Studies that used unvalidated self-reported measures did not find associations between digital interventions and SB reductions [32, 33].

### Meta-analysis

**Fig. 2:**
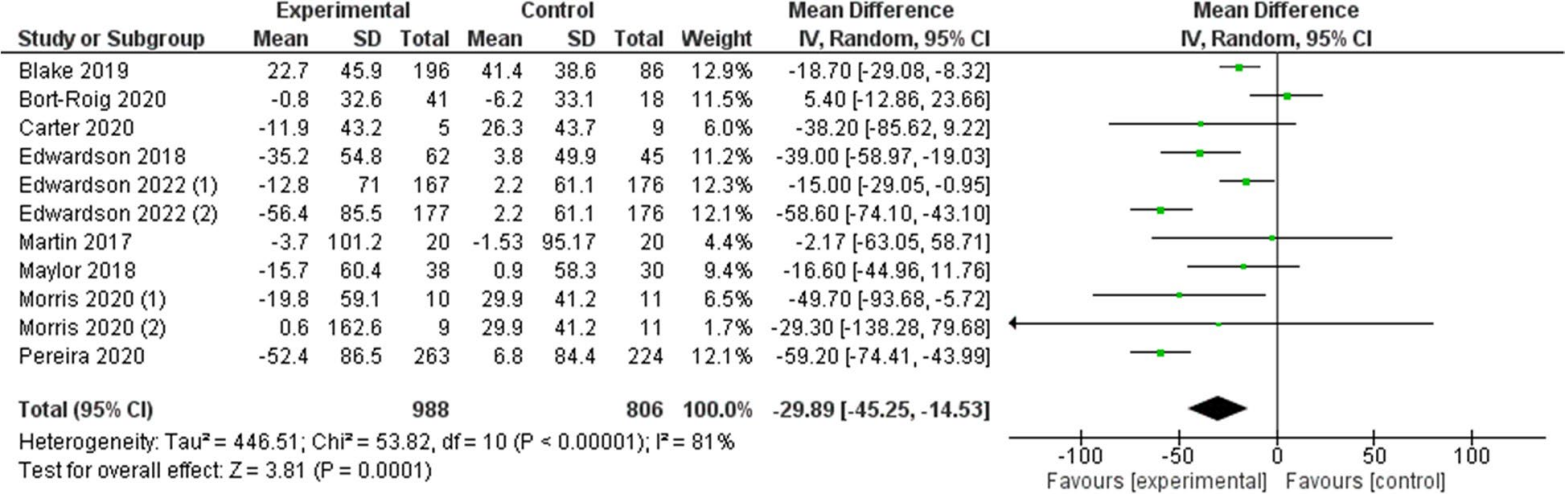
Total sedentary behaviour reductions (min/8 h workday)

Nine of the 19 studies were included in the meta-analysis [23–26, 29, 33, 35, 38, 40]. Two of the nine studies were considered as two independent studies due to the inclusion of three intervention arms [25, 40]. The reason for exclusion of the eight other studies was missing data (see Fig. 2).

The total change in workplace SB was -29.9 (95% CI: -45.3, -14.5) min/8 h workday (Z = 3.81; I^2^ = 81%) (see Fig. 2). The leave-one-out sensitivity analysis showed that the strength of the pooled estimate was robust and did not significantly differ when one study was omitted at a time (see Additional file 1). No changes in the pool estimated and confidence intervals were significant by exclusion of any one study. Removing the largest study [33] did not substantially change the point estimate (-31.4 (95% CI: -49.5, 13.4) min/8 h workday).

**Fig. 3:**
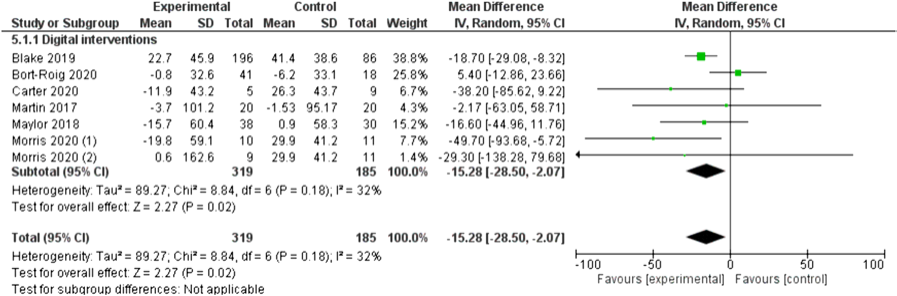
Digital interventions (min/8 h workday)

Figure 3 shows the results from the digital interventions subgroup, which covers interventions that were entirely digital interventions [29, 33, 35, 40], or digital interventions that included a unique non-digital element (i.e., an educational session) [26]. In this subgroup, SB was reduced by 15.28 (95% CI: -28.5, -2.07) min/8 h workday.

**Fig. 4:**
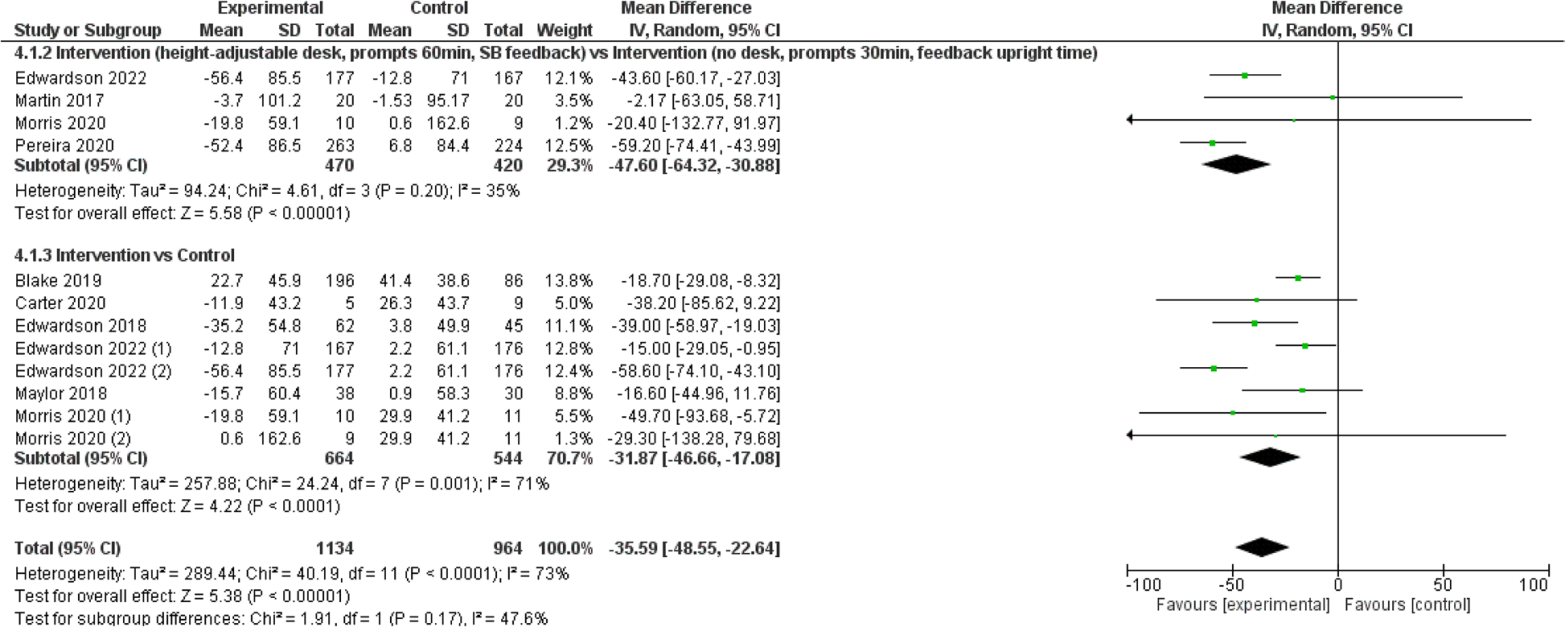
Sub-analysis sedentary behaviour reductions (min/8 h workday)

Figure 4 illustrates the pooled results from the sub-analysis. Four studies comprised the intervention vs intervention group [23, 25, 35, 40], eight studies comprised the intervention vs control [24–26, 33, 38, 40], and four of them belonged to two studies [25, 40]. Intervention arm subgroups identified a change of -35.6 (95% CI: -48.6, -22.6) min/8 h workday in SB.

**Fig. 5:**
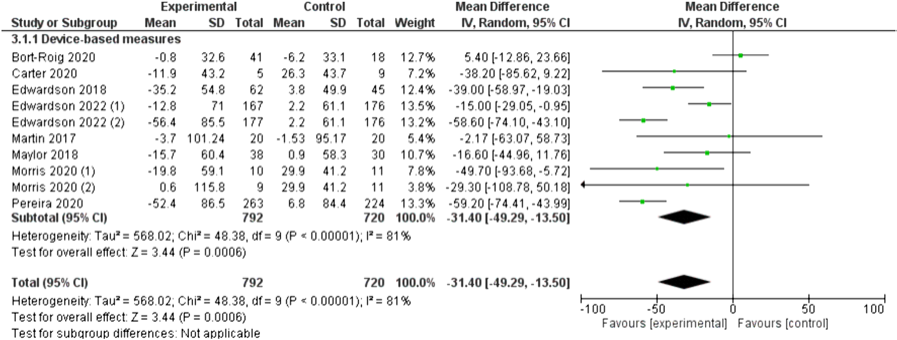
Device-based measures of sedentary behaviour reductions (min/8 h workday)

The results of the device-based measures subgroup are presented in Fig. 5, which includes 10 studies [23–26, 29, 35, 38, 40], four of which correspond to two studies [25, 40]. In this subgroup analysis, changes of -31.4 (95% CI: -49.3, -13.5) min/8 h workday were observed in SB.

### Risk of bias assessment

The mean quality score for 19 articles was 74.3%, ranging from 50% [36] to 92.9% [24]. The main reasons for lower scores were the lack of blinding of investigators and subjects (21.1% and 39.5%, respectively), and small sample sizes (44.7%). The higher scores included appropriate study design to respond to research questions and described and presented appropriate analysis (100%). The detailed quality score for each study can be found in Additional file 2.

## Discussion

The aim of this systematic review and meta-analysis was to explore the effectiveness of workplace interventions that incorporated digital elements to reduce SB in office workers. A total of 19 studies published between 2017 and 2023 met the inclusion criteria. In the identified studies, the most effective interventions were multicomponent and included a wide variety of digital features, with the delivery of information and educational materials the most common, followed by scheduled prompts to break SB or participate in PA and behaviour feedback. Text messages, e-newsletters, websites, and videos were the most common way to deliver information for increasing knowledge and awareness, while computer screens and mobile phone apps were the most typical way to deliver visual prompts.

Our meta-analysis highlights that workplace interventions that include digital elements (ranging from 8 weeks to 12 months) reduced SB by an average of 30 min/8 h workday, which is similar to a previous meta-analysis, demonstrating a reduction of 32.6 min/8 h workday [42], and slightly lower compared to other two meta-analyses with 40 min/8 h workday and 41 min/day [19] reductions. Two of these meta-analyses included studies with digital elements, although they did not focus on them in their analyses, combining the results of multiple intervention arms and time points into a standardised single result or included non-RCTs, which may indicate its higher result [16, 42]. The other study considered computer, mobile and wearable technology interventions to reduce SB across the whole day and the results were presented in minutes per day, which would explain our lower reductions presented in minutes per 8 h of workday [19]. The intervention effects seen in the present study may be clinically relevant, with evidence showing that a decrease in SB of 30 min or more per day had a favourable effect on body weight, body mass index, as well as significantly increased energy and social functioning and reduced pain and sleep disturbance [43, 44]. Additionally, replacing SB time of 30 min per day with low intensity PA or moderate-to-vigorous PA was associated with lower all-cause mortality risk [45], and reduced blood cholesterol [46].

The World Health Organisation (WHO) recommends breaking and limiting the time spent in SB in any context, including work, and replacing it with PA [47]. Although performing PA breaks involves working time, productivity is not affected, in fact it improves by improving other outcomes, such as health [48, 49]. This suggests that the use of technology, such as activity trackers and mobile phone applications, has great potential for measuring and encouraging PA [50] and has been shown to be effective in behavioural change interventions [19, 20] since these digital elements, aimed at health and PA, incorporate established behaviour change techniques [50]. Furthermore, digital elements may provide a crucial intervention tool as it provides information such as self-monitoring progress, individual goal progress, and real-time information at low cost, and usually is an acceptable tool according to workers’ opinions [20, 50]. Hence, our findings may show that technology is a great element to fulfil WHO recommendations, specifically in the workplace, where workers spend the most of their SB time.

Multicomponent interventions with two groups (i.e., intervention, and control groups) were the most represented among the studies, followed by information and counselling interventions. There was no representation of interventions only including physical changes in the workplace design and environment, and policies to change the organisation of work as intervention techniques alone. Our results of the meta-analysis suggest that multicomponent interventions including environmental changes (e.g. sit-stand desks) as the core element of the interventions, but were complemented by digital elements, reported the highest SB reductions (-59.2 (95% CI: -74.4, -44.) and -58.6 (95% CI: -74.1, -43.1)) [23, 25]. Interventions with environmental changes as core elements in the intervention have been shown to reduce SB and increase standing time, but not PA time. In addition, they showed difficulties in maintaining utilisation over time [51, 52]. Digital multicomponent interventions which only include digital elements, show the higher reductions in SB present prompts as the core component of the interventions (-49.7 (95% CI: -93.7, -5.72) and -38.2 (95% CI: -85.6, 9.22)) [38, 40]. Therefore, digital elements, such as prompts, may complement interventions with physical changes for maintaining and encouraging its use. Although the evidence shows the benefits of breaking SB time at work on health and work-related outcomes, the frequency and duration of the breaks are uncertain [53, 54]. Hence, future research should examine the most effective duration and frequency of SB breaks to reduce that behaviour.

Despite reductions in SB, multicomponent interventions, given their nature, have a large heterogeneity in the intervention’s components, as well as in the digital elements, making it difficult to compare them to determine the most effective intervention. Due to the lack of data, it was impossible in our meta-analysis to compare specific intervention types. Even though there is no conclusive evidence about the effectiveness of multicomponent interventions, the literature indicates that multicomponent interventions based on behavioural change theories, such as the BCW, theory of planned behaviour, and the socioecological model tend to be more effective [55]. Our results of the systematic review and meta-analysis suggest that interventions based on theories, including organisational strategies or policy components, environmental changes and educational or informational material reported higher SB reductions (-59.2 (95% CI: -74.4, -44.0) and -58.6 (-74.1, -43.1) min/8 h workday) [23, 25], than studies that have not been based on theories (5.4 (95% CI: -12.9, 23.7), -2.17 (95% CI: -63.1, 58.7), and -16.6 (95% CI: -45.0, 11.8) min/8 h workday) [26, 29, 35]. This finding may contribute to a better understanding of what components a behaviour change intervention should include to be effective.

The advancement of wearable technologies has made possible the device-based determination of activities based on body posture. The studies included in the present systematic review mainly reported the time spent in SB using device-based measures, especially through the activPAL device, which showed significant reductions of -31.4 (95% CI: -49.3, -13.5) min/8 h workday. Given the heterogeneity in unit measurement and the lack of data, effectiveness was not compared with other measurement tools, but evidence suggests that thigh-worn devices showed higher levels of accuracy to measure SB compared with wrist-worn devices [56]. Furthermore, self-report tools showed low correlation with device-based data and low precision [57]. A previous meta-analysis showed smaller reductions in time spent in SB for self-reported measures than device-based measures [16], which may be due to difficulties in recalling this behaviour, and therefore the difficulty to recollect the data accurately. These smaller reductions may be likely a result of the measurement method, rather than the intervention.

### Strengths and limitations

A key strength of this study is that, to our knowledge, it is the first systematic review that comprehensively assesses how workplace interventions that incorporated digital elements, affect office workers’ SB reductions, who are the most sedentary work sectors. Additionally, it is the first study that quantifies these findings through a meta-analysis and sub-analysis and present a mid-high quality (74.3%) of the included studies. However, the study includes acceptability and feasibility studies, as well as pilot studies presenting small sample sizes, lack of control of confounding and the lack of the assessment of statistically significant changes in the results.

This study has several limitations. One such limitation was the lack of opportunity to assess one intervention, using digital elements in one group and non-digital elements in the other group, to examine the effectiveness of the digital elements in the workplace interventions. The variety in SB unit measurement was a limitation of the current study. We standardised all the data to min/8-h workdays for the meta-analysis. That fact may have influenced our results, given lower reductions since not knowing whether total SB in the studies covered all day or only waking hours, we transformed the data from 24 to 8 h. The lack of data (i.e., mean differences from baseline to postintervention) and the nonresponse from the authors were other limitations for the meta-analysis, as the absence of data resulted in the removal of some studies. Overall, the meta-analysis showed greater heterogeneity (Chi^2^ = 53.82; I^2^ = 81%); hence, caution should be taken when interpreting these results.

### Future implications

Although the evidence supports the effectiveness of workplace interventions using digital elements in reducing SB in the traditional office setting, the hybrid work model (i.e., work in office and home) has become the customary mode of working for many employees since the COVID-19 pandemic [58]. This new paradigm of work has been associated with even more drastic increases in SB patterns [59, 60]. Therefore, future research should prioritise exploring how these theory-driven digital-based interventions, can be feasible for breaking and limiting SB when working from home. Additionally, it is important to investigate the adoption and maintenance of this behaviour change on employees' health and work performance. Recent evidence has identified digital interventions as complex interventions [20], and it is recommended to involve multiple stakeholders in the development process of these interventions to ensure their effectiveness in future studies [20, 61].

## Conclusions

This review provided evidence for the effectiveness of workplace interventions using digital elements to reduce SB among office workers. Our findings indicated an approximate reduction of 30 min per 8-h work day, suggesting that multicomponent interventions incorporating a wide variety of technological features (i.e., information delivery and mediated organisational support and social influences) may be effective approaches to reduce SB in workplaces. Considering the emerging evidence indicating an increase in SB in the hybrid work mode, future studies need to adapt these interventions in the home-office environment to evaluate their feasibility and effectiveness.

### Supplementary Information

**Additional file 1.** Leave-one-out. The document shows the influence of each study on the overall effect-size estimate trough figures excluding every time one study.

**Additional file 2.** Risk of bias assessment. The file shows on the first page the completed checklist of the QUALSYST for each study included in the systematic review. On the second page, the file presents the score of every item.

## Data Availability

All data generated or analysed during this study are included in this published article [and Additional files 1 and 2].

## Abbreviations

SB: Sedentary Behaviour
PA: Physical Activity
PRISMA: Preferred Reporting Items for Systematic reviews and Meta-Analyses
RCT: Randomised Controlled Trial
PICO: Population, Intervention, Comparison and Outcomes
AMD: Adjusted Mean Differences
SD: Standard Deviations
CI: Confidence Interval
SE: Standard Error
UMD: Unadjusted Mean Difference
GPAQ: Global Physical Activity Questionnaire
WSQ: Workforce Sitting Questionnaire
IPAQ: International Physical Activity Questionnaire
OSPAQ: Occupational Sitting and Physical Activity Questionnaire
WHO: World Health Organization
BCW: Behaviour Change Wheel
