# Supplementary material for: Effectiveness of workplace interventions with digital elements to reduce sedentary behaviours in office employees: a systematic review and meta-analysis
Source: Int J Behav Nutr Phys Act. 2024 Apr 19;21:41. doi: 10.1186/s12966-024-01595-6 (PMC11031993; doi:10.1186/s12966-024-01595-6)

Figure 2. Total sedentary behaviour reductions (min/8h workday)

	Experimental			Control				Mean Difference
Study or Subgroup	Mean	SD	Total	Mean	SD	Total	Weight	IV, Random, 95% CI
Blake 2019	22.7	45.9	196	41.4	38.6	86	12.9%	-18.70 [-29.08, -8.32]
Bort-Roig 2020	-0.8	32.6	41	-6.2	33.1	18	11.5%	5.40 [-12.86, 23.66]
Carter 2020	-11.9	43.2	5	26.3	43.7	9	6.0%	-38.20 [-85.62, 9.22]
Edwardson 2018	-35.2	54.8	62	3.8	49.9	45	11.2%	-39.00 [-58.97, -19.03]
Edwardson 2022 (1)	-12.8	71	167	2.2	61.1	176	12.3%	-15.00 [-29.05, -0.95]
Edwardson 2022 (2)	-56.4	85.5	177	2.2	61.1	176	12.1%	-58.60 [-74.10, -43.10]
Martin 2017	-3.7	101.2	20	-1.53	95.17	20	4.4%	-2.17 [-63.05, 58.71]
Maylor 2018	-15.7	60.4	38	0.9	58.3	30	9.4%	-16.60 [-44.96, 11.76]
Morris 2020 (1)	-19.8	59.1	10	29.9	41.2	11	6.5%	-49.70 [-93.68, -5.72]
Morris 2020 (2)	0.6	162.6	9	29.9	41.2	11	1.7%	-29.30 [-138.28, 79.68]
Pereira 2020	-52.4	86.5	263	6.8	84.4	224	12.1%	-59.20 [-74.41, -43.99]
**Total (95% CI)**			**988**			**806**	**100.0%**	**-29.89 [-45.25, -14.53]**

Heterogeneity: Tau² = 446.51; Chi² = 53.82, df = 10 (P < 0.00001); I² = 81%
Test for overall effect: Z = 3.81 (P = 0.0001)

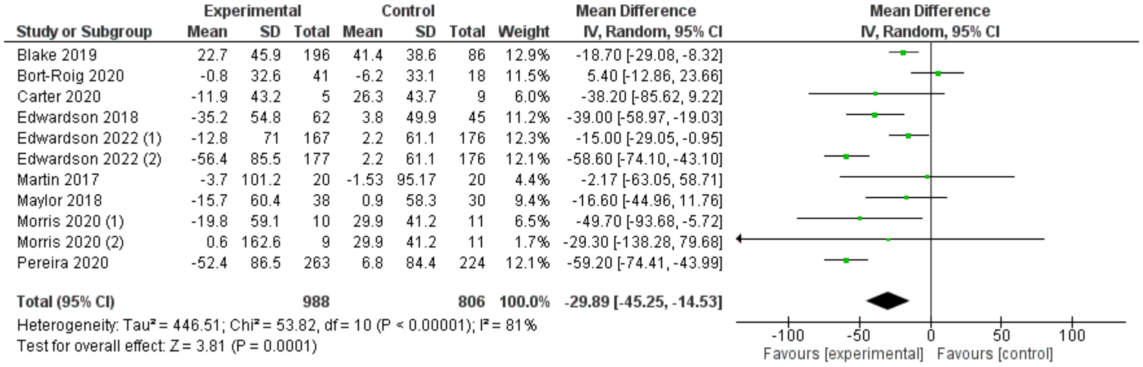

Figure 3. Leave-one-out (Blake 2019)

	Experimental			Control				Mean Difference
Study or Subgroup	Mean	SD	Total	Mean	SD	Total	Weight	IV, Random, 95% CI
Blake 2019	22.7	45.9	196	41.4	38.6	86	0.0%	-18.70 [-29.08, -8.32]
Bort-Roig 2020	-0.8	32.6	41	-6.2	33.1	18	12.9%	5.40 [-12.86, 23.66]
Carter 2020	-11.9	43.2	5	26.3	43.7	9	7.3%	-38.20 [-85.62, 9.22]
Edwardson 2018	-35.2	54.8	62	3.8	49.9	45	12.6%	-39.00 [-58.97, -19.03]
Edwardson 2022 (1)	-12.8	71	167	2.2	61.1	176	13.6%	-15.00 [-29.05, -0.95]
Edwardson 2022 (2)	-56.4	85.5	177	2.2	61.1	176	13.4%	-58.60 [-74.10, -43.10]
Martin 2017	-3.7	101.2	20	-1.53	95.17	20	5.5%	-2.17 [-63.05, 58.71]
Maylor 2018	-15.7	60.4	38	0.9	58.3	30	10.9%	-16.60 [-44.96, 11.76]
Morris 2020 (1)	-19.8	59.1	10	29.9	41.2	11	7.9%	-49.70 [-93.68, -5.72]
Morris 2020 (2)	0.6	162.6	9	29.9	41.2	11	2.3%	-29.30 [-138.28, 79.68]
Pereira 2020	-52.4	86.5	263	6.8	84.4	224	13.5%	-59.20 [-74.41, -43.99]
**Total (95% CI)**			**792**			**720**	**100.0%**	**-31.42 [-49.48, -13.36]**

Heterogeneity: Tau² = 570.67; Chi² = 48.37, df = 9 (P < 0.00001); I² = 81%
Test for overall effect: Z = 3.41 (P = 0.0006)

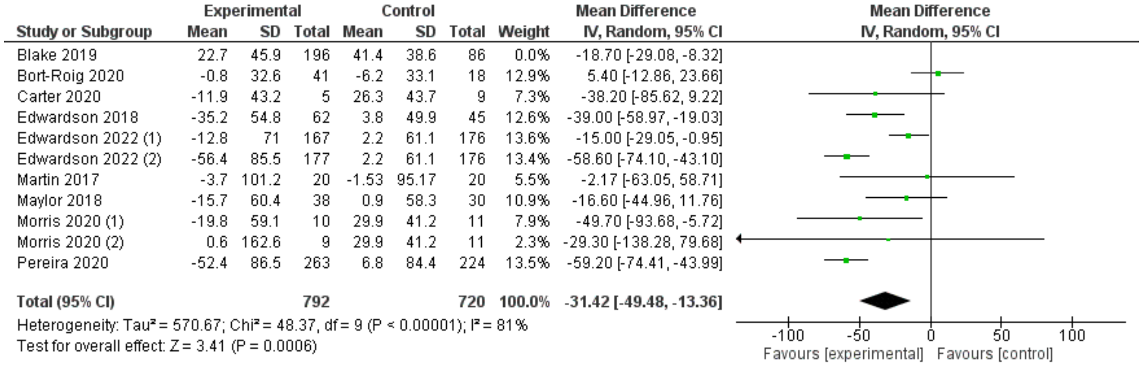

Figure 4. Leave.one-out (Bort-Roig 2020)

	Experimental			Control				Mean Difference
Study or Subgroup	Mean	SD	Total	Mean	SD	Total	Weight	IV, Random, 95% CI
Blake 2019	22.7	45.9	196	41.4	38.6	86	15.3%	-18.70 [-29.08, -8.32]
Bort-Roig 2020	-0.8	32.6	41	-6.2	33.1	18	0.0%	5.40 [-12.86, 23.66]
Carter 2020	-11.9	43.2	5	26.3	43.7	9	6.2%	-38.20 [-85.62, 9.22]
Edwardson 2018	-35.2	54.8	62	3.8	49.9	45	12.8%	-39.00 [-58.97, -19.03]
Edwardson 2022 (1)	-12.8	71	167	2.2	61.1	176	14.4%	-15.00 [-29.05, -0.95]
Edwardson 2022 (2)	-56.4	85.5	177	2.2	61.1	176	14.0%	-58.60 [-74.10, -43.10]
Martin 2017	-3.7	101.2	20	-1.53	95.17	20	4.4%	-2.17 [-63.05, 58.71]
Maylor 2018	-15.7	60.4	38	0.9	58.3	30	10.3%	-16.60 [-44.96, 11.76]
Morris 2020 (1)	-19.8	59.1	10	29.9	41.2	11	6.8%	-49.70 [-93.68, -5.72]
Morris 2020 (2)	0.6	162.6	9	29.9	41.2	11	1.7%	-29.30 [-138.28, 79.68]
Pereira 2020	-52.4	86.5	263	6.8	84.4	224	14.1%	-59.20 [-74.41, -43.99]
**Total (95% CI)**			**947**			**788**	**100.0%**	**-34.61 [-49.47, -19.76]**

Heterogeneity: Tau² = 346.52; Chi² = 38.68, df = 9 (P < 0.0001); I² = 77%
Test for overall effect: Z = 4.57 (P < 0.00001)

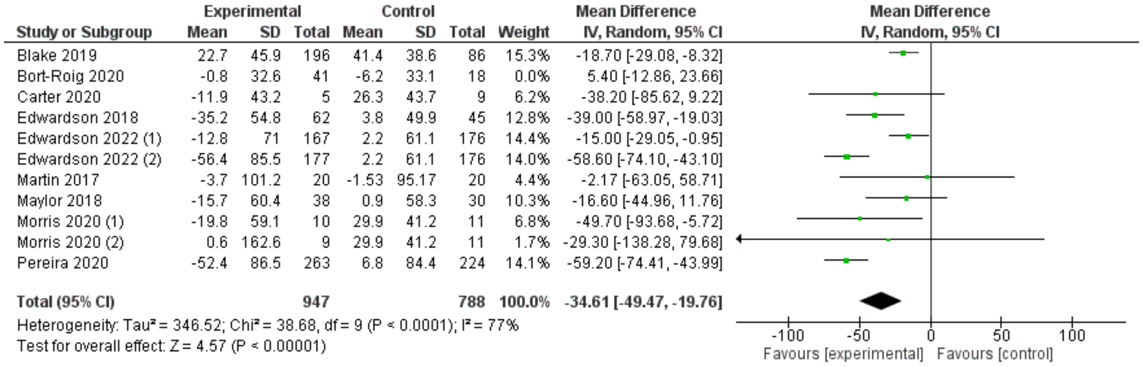

Figure 5. Leave-one-out (Carter 2020)

	Experimental			Control				Mean Difference
Study or Subgroup	Mean	SD	Total	Mean	SD	Total	Weight	IV, Random, 95% CI
Blake 2019	22.7	45.9	196	41.4	38.6	86	13.7%	-18.70 [-29.08, -8.32]
Bort-Roig 2020	-0.8	32.6	41	-6.2	33.1	18	12.2%	5.40 [-12.86, 23.66]
Carter 2020	-11.9	43.2	5	26.3	43.7	9	0.0%	-38.20 [-85.62, 9.22]
Edwardson 2018	-35.2	54.8	62	3.8	49.9	45	11.9%	-39.00 [-58.97, -19.03]
Edwardson 2022 (1)	-12.8	71	167	2.2	61.1	176	13.1%	-15.00 [-29.05, -0.95]
Edwardson 2022 (2)	-56.4	85.5	177	2.2	61.1	176	12.8%	-58.60 [-74.10, -43.10]
Martin 2017	-3.7	101.2	20	-1.53	95.17	20	4.7%	-2.17 [-63.05, 58.71]
Maylor 2018	-15.7	60.4	38	0.9	58.3	30	10.0%	-16.60 [-44.96, 11.76]
Morris 2020 (1)	-19.8	59.1	10	29.9	41.2	11	7.0%	-49.70 [-93.68, -5.72]
Morris 2020 (2)	0.6	162.6	9	29.9	41.2	11	1.9%	-29.30 [-138.28, 79.68]
Pereira 2020	-52.4	86.5	263	6.8	84.4	224	12.8%	-59.20 [-74.41, -43.99]
**Total (95% CI)**			**983**			**797**	**100.0%**	**-29.35 [-45.44, -13.26]**

Heterogeneity: Tau² = 464.61; Chi² = 53.68, df = 9 (P < 0.00001); I² = 83%
Test for overall effect: Z = 3.58 (P = 0.0004)

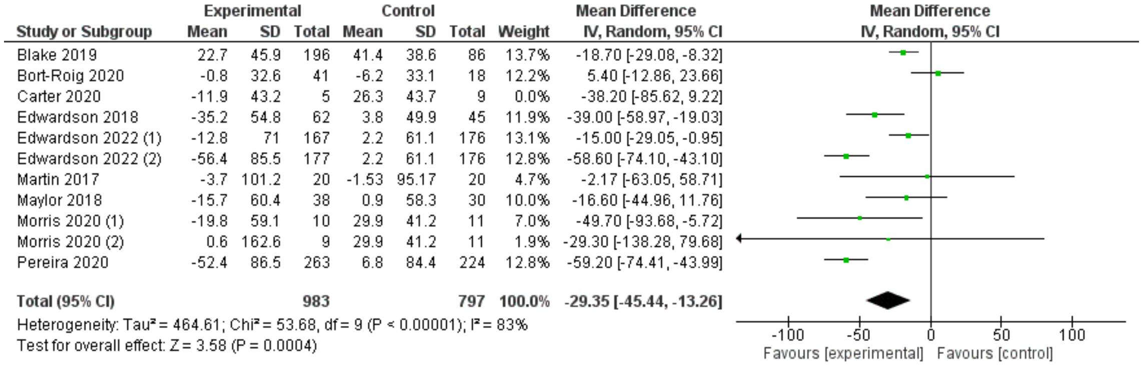

Figure 6. Leave-one-out (Edwardson 2018)

Study or Subgroup	Experimental Mean	Experimental SD	Experimental Total	Control Mean	Control SD	Control Total	Weight	Mean Difference IV, Random, 95% CI
Blake 2019	22.7	45.9	196	41.4	38.6	86	14.3%	-18.70 [-29.08, -8.32]
Bort-Roig 2020	-0.8	32.6	41	-6.2	33.1	18	12.8%	5.40 [-12.86, 23.66]
Carter 2020	-11.9	43.2	5	26.3	43.7	9	6.9%	-38.20 [-85.62, 9.22]
Edwardson 2018	-35.2	54.8	62	3.8	49.9	45	0.0%	-39.00 [-58.97, -19.03]
Edwardson 2022 (1)	-12.8	71	167	2.2	61.1	176	13.7%	-15.00 [-29.05, -0.95]
Edwardson 2022 (2)	-56.4	85.5	177	2.2	61.1	176	13.4%	-58.60 [-74.10, -43.10]
Martin 2017	-3.7	101.2	20	-1.53	95.17	20	5.1%	-2.17 [-63.05, 58.71]
Maylor 2018	-15.7	60.4	38	0.9	58.3	30	10.6%	-16.60 [-44.96, 11.76]
Morris 2020 (1)	-19.8	59.1	10	29.9	41.2	11	7.5%	-49.70 [-93.68, -5.72]
Morris 2020 (2)	0.6	162.6	9	29.9	41.2	11	2.1%	-29.30 [-138.28, 79.68]
Pereira 2020	-52.4	86.5	263	6.8	84.4	224	13.5%	-59.20 [-74.41, -43.99]
**Total (95% CI)**			**926**			**761**	**100.0%**	**-28.73 [-45.75, -11.71]**

Heterogeneity: $Tau^2 = 500.12$; $Chi^2 = 52.78$, df = 9 ($P < 0.00001$); $I^2 = 83\%$
Test for overall effect: Z = 3.31 (P = 0.0009)

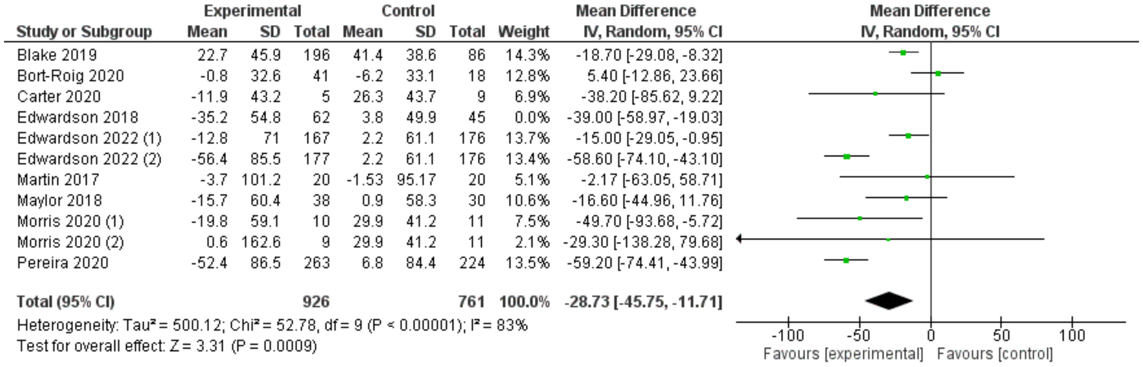

Figure 7. Leave-one-out (Edwardson 2022-1)

Study or Subgroup	Experimental Mean	Experimental SD	Experimental Total	Control Mean	Control SD	Control Total	Weight	Mean Difference IV, Random, 95% CI
Blake 2019	22.7	45.9	196	41.4	38.6	86	14.4%	-18.70 [-29.08, -8.32]
Bort-Roig 2020	-0.8	32.6	41	-6.2	33.1	18	13.0%	5.40 [-12.86, 23.66]
Carter 2020	-11.9	43.2	5	26.3	43.7	9	7.1%	-38.20 [-85.62, 9.22]
Edwardson 2018	-35.2	54.8	62	3.8	49.9	45	12.6%	-39.00 [-58.97, -19.03]
Edwardson 2022 (1)	-12.8	71	167	2.2	61.1	176	0.0%	-15.00 [-29.05, -0.95]
Edwardson 2022 (2)	-56.4	85.5	177	2.2	61.1	176	13.5%	-58.60 [-74.10, -43.10]
Martin 2017	-3.7	101.2	20	-1.53	95.17	20	5.3%	-2.17 [-63.05, 58.71]
Maylor 2018	-15.7	60.4	38	0.9	58.3	30	10.8%	-16.60 [-44.96, 11.76]
Morris 2020 (1)	-19.8	59.1	10	29.9	41.2	11	7.7%	-49.70 [-93.68, -5.72]
Morris 2020 (2)	0.6	162.6	9	29.9	41.2	11	2.2%	-29.30 [-138.28, 79.68]
Pereira 2020	-52.4	86.5	263	6.8	84.4	224	13.6%	-59.20 [-74.41, -43.99]
**Total (95% CI)**			**821**			**630**	**100.0%**	**-31.91 [-49.20, -14.63]**

Heterogeneity: $Tau^2 = 512.65$; $Chi^2 = 49.23$, df = 9 ($P < 0.00001$); $I^2 = 82\%$
Test for overall effect: Z = 3.62 (P = 0.0003)

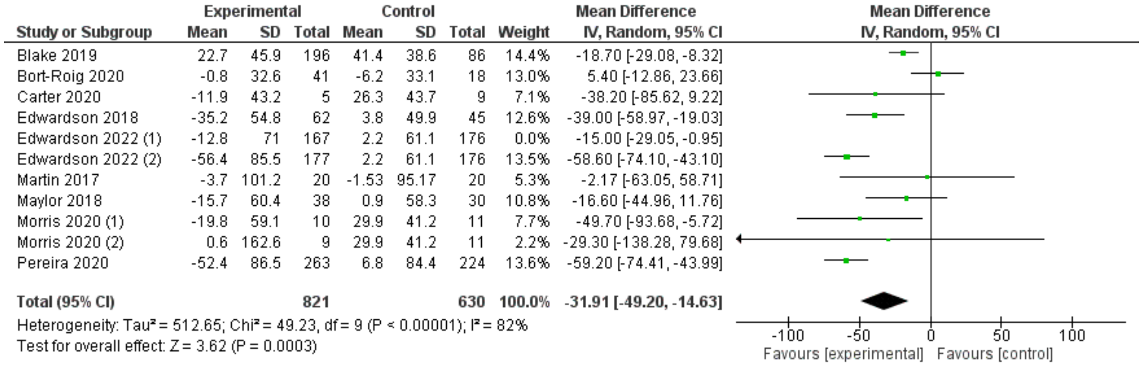

Figure 8. Leave-one-out (Edwardson 2022-2)

Study or Subgroup	Experimental Mean	Experimental SD	Experimental Total	Control Mean	Control SD	Control Total	Weight	Mean Difference IV, Random, 95% CI
Blake 2019	22.7	45.9	196	41.4	38.6	86	15.4%	-18.70 [-29.08, -8.32]
Bort-Roig 2020	-0.8	32.6	41	-6.2	33.1	18	13.3%	5.40 [-12.86, 23.66]
Carter 2020	-11.9	43.2	5	26.3	43.7	9	6.2%	-38.20 [-85.62, 9.22]
Edwardson 2018	-35.2	54.8	62	3.8	49.9	45	12.8%	-39.00 [-58.97, -19.03]
Edwardson 2022 (1)	-12.8	71	167	2.2	61.1	176	14.5%	-15.00 [-29.05, -0.95]
Edwardson 2022 (2)	-56.4	85.5	177	2.2	61.1	176	0.0%	-58.60 [-74.10, -43.10]
Martin 2017	-3.7	101.2	20	-1.53	95.17	20	4.4%	-2.17 [-63.05, 58.71]
Maylor 2018	-15.7	60.4	38	0.9	58.3	30	10.4%	-16.60 [-44.96, 11.76]
Morris 2020 (1)	-19.8	59.1	10	29.9	41.2	11	6.8%	-49.70 [-93.68, -5.72]
Morris 2020 (2)	0.6	162.6	9	29.9	41.2	11	1.7%	-29.30 [-138.28, 79.68]
Pereira 2020	-52.4	86.5	263	6.8	84.4	224	14.2%	-59.20 [-74.41, -43.99]
**Total (95% CI)**			**811**			**630**	**100.0%**	**-25.87 [-40.87, -10.87]**

Heterogeneity: $Tau^2 = 352.05$; $Chi^2 = 37.67$, df = 9 ($P < 0.0001$); $I^2 = 76\%$
Test for overall effect: Z = 3.38 (P = 0.0007)

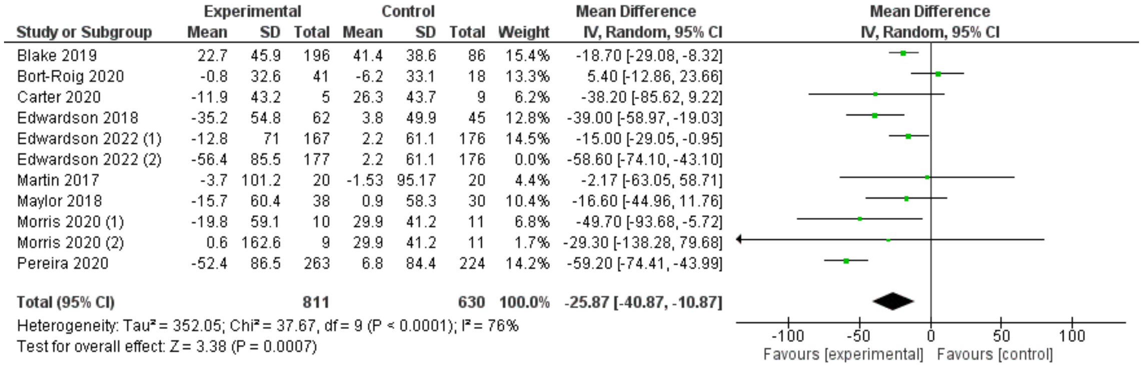

Figure 9. Leave-one-out (Martin 2017)

	Experimental			Control				Mean Difference
Study or Subgroup	Mean	SD	Total	Mean	SD	Total	Weight	IV, Random, 95% CI
Blake 2019	22.7	45.9	196	41.4	38.6	86	13.5%	-18.70 [-29.08, -8.32]
Bort-Roig 2020	-0.8	32.6	41	-6.2	33.1	18	12.0%	5.40 [-12.86, 23.66]
Carter 2020	-11.9	43.2	5	26.3	43.7	9	6.3%	-38.20 [-85.62, 9.22]
Edwardson 2018	-35.2	54.8	62	3.8	49.9	45	11.7%	-39.00 [-58.97, -19.03]
Edwardson 2022 (1)	-12.8	71	167	2.2	61.1	176	12.9%	-15.00 [-29.05, -0.95]
Edwardson 2022 (2)	-56.4	85.5	177	2.2	61.1	176	12.6%	-58.60 [-74.10, -43.10]
Martin 2017	-3.7	101.2	20	-1.53	95.17	20	0.0%	-2.17 [-63.05, 58.71]
Maylor 2018	-15.7	60.4	38	0.9	58.3	30	9.8%	-16.60 [-44.96, 11.76]
Morris 2020 (1)	-19.8	59.1	10	29.9	41.2	11	6.8%	-49.70 [-93.68, -5.72]
Morris 2020 (2)	0.6	162.6	9	29.9	41.2	11	1.8%	-29.30 [-138.28, 79.68]
Pereira 2020	-52.4	86.5	263	6.8	84.4	224	12.6%	-59.20 [-74.41, -43.99]
**Total (95% CI)**			**968**			**786**	**100.0%**	**-31.16 [-46.97, -15.34]**

Heterogeneity: Tau² = 454.60; Chi² = 53.07, df = 9 (P < 0.00001); I² = 83%
Test for overall effect: Z = 3.86 (P = 0.0001)

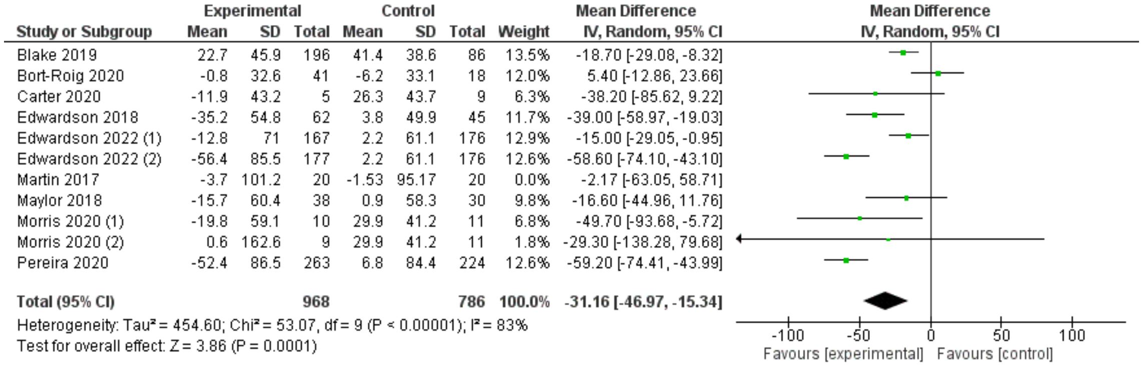

Figure 10. Leave-one-out (Maylor 2018)

	Experimental			Control				Mean Difference
Study or Subgroup	Mean	SD	Total	Mean	SD	Total	Weight	IV, Random, 95% CI
Blake 2019	22.7	45.9	196	41.4	38.6	86	14.1%	-18.70 [-29.08, -8.32]
Bort-Roig 2020	-0.8	32.6	41	-6.2	33.1	18	12.7%	5.40 [-12.86, 23.66]
Carter 2020	-11.9	43.2	5	26.3	43.7	9	6.7%	-38.20 [-85.62, 9.22]
Edwardson 2018	-35.2	54.8	62	3.8	49.9	45	12.3%	-39.00 [-58.97, -19.03]
Edwardson 2022 (1)	-12.8	71	167	2.2	61.1	176	13.5%	-15.00 [-29.05, -0.95]
Edwardson 2022 (2)	-56.4	85.5	177	2.2	61.1	176	13.2%	-58.60 [-74.10, -43.10]
Martin 2017	-3.7	101.2	20	-1.53	95.17	20	4.9%	-2.17 [-63.05, 58.71]
Maylor 2018	-15.7	60.4	38	0.9	58.3	30	0.0%	-16.60 [-44.96, 11.76]
Morris 2020 (1)	-19.8	59.1	10	29.9	41.2	11	7.3%	-49.70 [-93.68, -5.72]
Morris 2020 (2)	0.6	162.6	9	29.9	41.2	11	2.0%	-29.30 [-138.28, 79.68]
Pereira 2020	-52.4	86.5	263	6.8	84.4	224	13.3%	-59.20 [-74.41, -43.99]
**Total (95% CI)**			**950**			**776**	**100.0%**	**-31.26 [-47.79, -14.73]**

Heterogeneity: Tau² = 475.18; Chi² = 53.05, df = 9 (P < 0.00001); I² = 83%
Test for overall effect: Z = 3.71 (P = 0.0002)

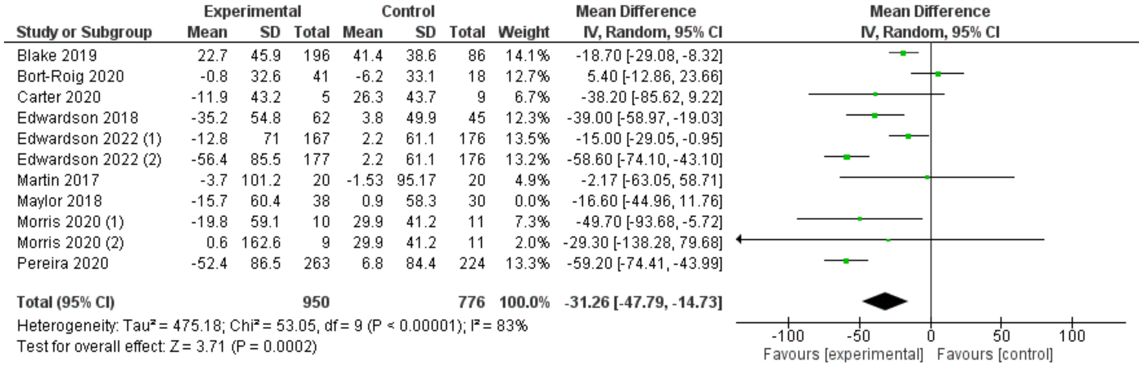

Figure 11. Leave-one-out (Morris 2020-1)

	Experimental			Control				Mean Difference
Study or Subgroup	Mean	SD	Total	Mean	SD	Total	Weight	IV, Random, 95% CI
Blake 2019	22.7	45.9	196	41.4	38.6	86	13.8%	-18.70 [-29.08, -8.32]
Bort-Roig 2020	-0.8	32.6	41	-6.2	33.1	18	12.3%	5.40 [-12.86, 23.66]
Carter 2020	-11.9	43.2	5	26.3	43.7	9	6.4%	-38.20 [-85.62, 9.22]
Edwardson 2018	-35.2	54.8	62	3.8	49.9	45	11.9%	-39.00 [-58.97, -19.03]
Edwardson 2022 (1)	-12.8	71	167	2.2	61.1	176	13.1%	-15.00 [-29.05, -0.95]
Edwardson 2022 (2)	-56.4	85.5	177	2.2	61.1	176	12.9%	-58.60 [-74.10, -43.10]
Martin 2017	-3.7	101.2	20	-1.53	95.17	20	4.7%	-2.17 [-63.05, 58.71]
Maylor 2018	-15.7	60.4	38	0.9	58.3	30	10.0%	-16.60 [-44.96, 11.76]
Morris 2020 (1)	-19.8	59.1	10	29.9	41.2	11	0.0%	-49.70 [-93.68, -5.72]
Morris 2020 (2)	0.6	162.6	9	29.9	41.2	11	1.9%	-29.30 [-138.28, 79.68]
Pereira 2020	-52.4	86.5	263	6.8	84.4	224	12.9%	-59.20 [-74.41, -43.99]
**Total (95% CI)**			**978**			**795**	**100.0%**	**-28.50 [-44.55, -12.45]**

Heterogeneity: Tau² = 458.68; Chi² = 52.96, df = 9 (P < 0.00001); I² = 83%
Test for overall effect: Z = 3.48 (P = 0.0005)

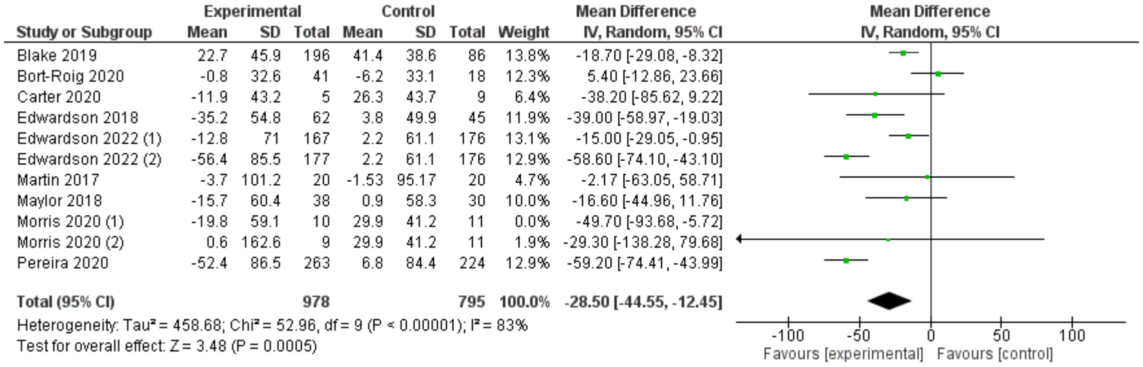

Figure 12. Leave-one-out (Morris 2020-2)

	Experimental			Control				Mean Difference
Study or Subgroup	Mean	SD	Total	Mean	SD	Total	Weight	IV, Random, 95% CI
Blake 2019	22.7	45.9	196	41.4	38.6	86	13.1%	-18.70 [-29.08, -8.32]
Bort-Roig 2020	-0.8	32.6	41	-6.2	33.1	18	11.7%	5.40 [-12.86, 23.66]
Carter 2020	-11.9	43.2	5	26.3	43.7	9	6.1%	-38.20 [-85.62, 9.22]
Edwardson 2018	-35.2	54.8	62	3.8	49.9	45	11.3%	-39.00 [-58.97, -19.03]
Edwardson 2022 (1)	-12.8	71	167	2.2	61.1	176	12.5%	-15.00 [-29.05, -0.95]
Edwardson 2022 (2)	-56.4	85.5	177	2.2	61.1	176	12.2%	-58.60 [-74.10, -43.10]
Martin 2017	-3.7	101.2	20	-1.53	95.17	20	4.5%	-2.17 [-63.05, 58.71]
Maylor 2018	-15.7	60.4	38	0.9	58.3	30	9.6%	-16.60 [-44.96, 11.76]
Morris 2020 (1)	-19.8	59.1	10	29.9	41.2	11	6.6%	-49.70 [-93.68, -5.72]
Morris 2020 (2)	0.6	162.6	9	29.9	41.2	11	0.0%	-29.30 [-138.28, 79.68]
Pereira 2020	-52.4	86.5	263	6.8	84.4	224	12.3%	-59.20 [-74.41, -43.99]
**Total (95% CI)**			**979**			**795**	**100.0%**	**-29.89 [-45.55, -14.24]**

Heterogeneity: Tau² = 458.47; Chi² = 53.82, df = 9 (P < 0.00001); I² = 83%
Test for overall effect: Z = 3.74 (P = 0.0002)

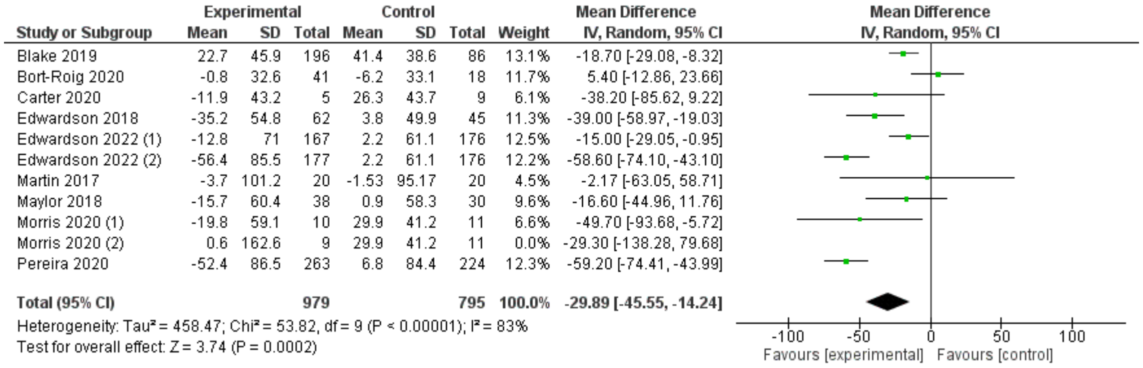

Figure 13. Leave-one-out (Pereira 2020)

	Experimental			Control				Mean Difference
Study or Subgroup	Mean	SD	Total	Mean	SD	Total	Weight	IV, Random, 95% CI
Blake 2019	22.7	45.9	196	41.4	38.6	86	15.6%	-18.70 [-29.08, -8.32]
Bort-Roig 2020	-0.8	32.6	41	-6.2	33.1	18	13.4%	5.40 [-12.86, 23.66]
Carter 2020	-11.9	43.2	5	26.3	43.7	9	6.2%	-38.20 [-85.62, 9.22]
Edwardson 2018	-35.2	54.8	62	3.8	49.9	45	12.9%	-39.00 [-58.97, -19.03]
Edwardson 2022 (1)	-12.8	71	167	2.2	61.1	176	14.6%	-15.00 [-29.05, -0.95]
Edwardson 2022 (2)	-56.4	85.5	177	2.2	61.1	176	14.2%	-58.60 [-74.10, -43.10]
Martin 2017	-3.7	101.2	20	-1.53	95.17	20	4.4%	-2.17 [-63.05, 58.71]
Maylor 2018	-15.7	60.4	38	0.9	58.3	30	10.4%	-16.60 [-44.96, 11.76]
Morris 2020 (1)	-19.8	59.1	10	29.9	41.2	11	6.8%	-49.70 [-93.68, -5.72]
Morris 2020 (2)	0.6	162.6	9	29.9	41.2	11	1.7%	-29.30 [-138.28, 79.68]
Pereira 2020	-52.4	86.5	263	6.8	84.4	224	0.0%	-59.20 [-74.41, -43.99]
**Total (95% CI)**			**725**			**582**	**100.0%**	**-25.75 [-40.52, -10.98]**

Heterogeneity: Tau² = 337.11; Chi² = 36.27, df = 9 (P < 0.0001); I² = 75%
Test for overall effect: Z = 3.42 (P = 0.0006)

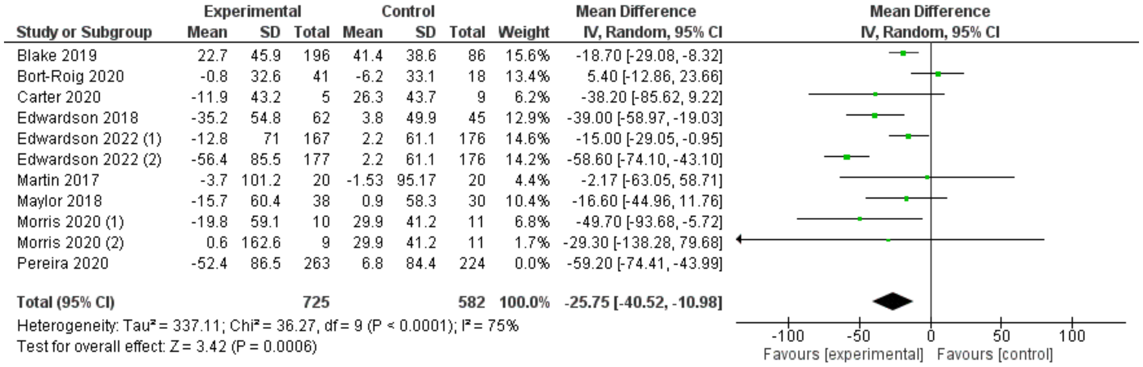

Supplement: Supplementary file 1 — Additional file 1. Leave-one-out. The document shows the influence of each study on the overall effect-size estimate trough figures excluding every time one study. [file 12966_2024_1595_MOESM1_ESM.pdf]
